# Effect of smoking on tuberculosis treatment outcomes: A systematic review and meta-analysis

**DOI:** 10.1371/journal.pone.0239333

**Published:** 2020-09-17

**Authors:** Abay Burusie, Fikre Enquesilassie, Adamu Addissie, Berhe Dessalegn, Tafesse Lamaro

**Affiliations:** 1 Department of Public Health, College of Health Sciences, Arsi University, Asella, Ethiopia; 2 School of Public Health, College of Health Sciences, Addis Ababa University, Addis Ababa, Ethiopia; 3 Department of Public Health, College of Health Sciences, Adigrat University, Adigrat, Ethiopia; 4 Department of Nursing, College of Health Sciences, Mizan-Tepi University, Tepi, Ethiopia; University of Calfornia San Francisco, UNITED STATES

## Abstract

**Introduction:**

Numerous studies have explored an effect of cigarette smoking on tuberculosis treatment outcomes but with dissimilar conclusions.

**Objective:**

To determine the effect of cigarette smoking on tuberculosis treatment outcomes.

**Methods:**

PubMed, Cochrane library and Google scholar databases were searched last on February 27, 2019. We applied the random-effects model for the analysis. Publication bias was assessed using funnel plot and Egger’s regression. Furthermore, we performed Orwin’s Fail-Safe N and cumulative meta-analysis to check for small studies’ effect.

**Results:**

Out of 22 studies we included in the qualitative synthesis, 12 studies reported p-values less than 0.05 where smoking significantly favored poor treatment outcomes. The remaining 10 studies reported p-values larger than 0.05 implying that smoking does not affect the treatment outcomes. Twenty studies met the criteria for inclusion in a meta-analysis. The meta-analysis found that smoking significantly increased the likelihood of poor tuberculosis treatment outcomes by 51% (OR = 1.51; 95% CI = 1.30 to 1.75 and I-square = 75.1%). In a sub-group analysis, the effect was higher for low- and middle-income countries (OR = 1.74; 95% CI = 1.31 to 2.30) and upper-middle-income economies (OR = 1.52; 95% CI = 1.16 to 1.98) than for high-income ones (OR = 1.34; 95% CI = 1.03 to 1.75) even though the differences in the effects among the strata were not statistically significant as demonstrated by overlapping of confidence intervals of the effects. Meta-regression analysis, adjusted for income economies, found the effect of smoking has not significantly improved over the years (p = 0.92) and thus implying neither of the covariates were source of the heterogeneity. Egger’s regression test indicated that publication bias is unlikely (p = 0.403).

**Conclusion:**

Cigarette smoking is significantly linked with poor tuberculosis treatment outcomes.

## Introduction

Tobacco is responsible for one in 10 deaths around the world [1] and the prevalence of smoking tobacco is declining slowly, especially in low- and middle-income countries [2]. The health effect of tobacco smoke is not limited to the smoker. Inhaling smoke as a passive smoker also substantially harms the health of second-hand smokers [3]. Smoking affects both innate and adaptive immunity in humans and thus weakens defensive immunity [4]. That seems the reason why smokers are also at increased risk for extra-pulmonary tuberculosis [5]. However, negative effects of cigarette smoking on the immune system subside within six weeks after smoking cessation [6, 7]. Studies indicate a high prevalence of smoking among tuberculosis patients [8–10]. Cigarette smoking increases the risk of infection by mycobacterium tuberculosis as well as the risk of TB disease development in the infected individuals [11]. Similarly, passive smoking or exposure to second-hand smoke is a risk factor for infection by mycobacterium tuberculosis and developing TB disease [12]. Moreover, meta-analysis studies report that poor or unfavorable tuberculosis treatment outcomes like mortality [13] and drug-resistant TB [14] are significantly associated with cigarette smoking. Smoking is also identified as an independent predictor of delayed or non-conversion of sputum culture, a sign of likely treatment failure [15, 16]. On the other hand, a separate meta-analysis concluded that smoking did not lead to risk of additional mortality in tuberculosis patients taking anti-TB treatment [11]. Thus, the need to conduct additional systematic reviews and meta-analyses continues where they can incorporate results from recently added studies until we obtain consistent conclusions. Furthermore, previous reviews of poor tuberculosis treatment outcomes especially in relation to tobacco were limited to assessing its role in mortality, treatment failure and non-conversion of sputum culture. However, we expanded this review to consider loss-to follow up as poor outcome in this meta-analysis because tobacco smoking causes a lack of adherence to anti-tuberculosis treatment [17, 18].

This review and meta-analysis aimed to assess the effect of cigarette smoking on tuberculosis treatment outcomes.

## Materials and methods

### Inclusion and exclusion

We defined inclusion criteria for articles to include in our search as including clinical trials, quasi-experimental, cohort, case-control and cross-sectional studies, that measured the effect of smoking on tuberculosis treatment outcomes. We limited our search to studies conducted on patients diagnosed with tuberculosis and who started anti-tuberculosis treatment and that included a comparison of TB patients who smoked or were exposed to smoking through second-hand smoke with patients who were non-smokers or had ceased smoking when starting anti-TB treatment. We did not include studies performed on patients treated surgically in addition to anti-TB drugs.

To be included in our analysis, study outcomes had to include the following tuberculosis treatment outcomes: died, treatment failure, or loss to follow up—categorized as poor outcomes—and patient cured or treatment completed—categorized as successful outcomes. We considered studies for the analysis when they measured TB treatment outcomes according to the WHO definitions [19] below:

**Died.** “A TB patient who dies for any reason before starting or during the course of treatment”;

**Treatment failure.** “A TB patient whose sputum smear or culture is positive at month five or later of treatment”;

**Loss to follow-up.** “A TB patient who did not start treatment or whose treatment was interrupted for two consecutive months or more”;

**Cured.** “A pulmonary TB patient with bacteriologically-confirmed TB at the beginning of treatment who was smear- or culture-negative in last month of treatment and on at least one previous occasion”;

**Treatment completed.** “A TB patient who completed treatment without evidence of failure, but with no record to show that sputum smear or culture results in the last month of treatment and on at least one previous occasion were negative, either because tests were not done or because results are unavailable”;

### Search strategy

We used the PICO acronym (**P**articipants; **I**nterventions/exposures; **C**omparators and **O**utcomes) as a guide to develop search terms for electronic database search. In this specific review;

- Participants in the studies were tuberculosis patients who were receiving anti-TB treatment
- The intervention was smoking cessation or no history of smoking by the participant
- The controls are tuberculosis patients who are smokers or have exposure to second-hand smoke
- Outcomes included any of the tuberculosis treatment outcomes defined above

First, we searched PubMed, the Cochrane library and google scholar databases using free-text and Medical Subject-Heading (MeSH) terms for tuberculosis, treatment outcome and smoking without restrictions as to study period. Thereafter, we undertook advanced searching by combining the search terms using Boolean operators. We concluded any electronic database search on February 27, 2019 (S1 Annex). However, we screened reference lists of studies extracted through the electronic search to identify additional studies.

### Screening of studies

We eliminated duplicate records using EndNote software. We screened titles and abstracts and removed records that did not meet our criteria, such as guidelines, reviews, position papers and those without an abstract (only titles available).

Two authors (A.B. and T.L) independently assessed the eligibility of full-text studies. In addition to the inclusion criteria for qualitative synthesis, studies were required to have frequencies for a 2x2 table or have sufficient information to extract these frequencies and enable computation of odds ratios for the meta-analysis. The authors resolved disagreements on the eligibility of a study by discussing and reaching consensus. However, we considered only studies written in English language.

### Data extraction

A.B. and B.D extracted the following data into an excel sheet: study author(s), publication year, country, study design, proportion of HIV positive participants, type of TB assessed (drug-susceptible or drug-resistant or mixed), proportion of participants aged less than 15 years, categories of TB treatment outcomes (died, treatment failure, loss to follow up, cured, treatment completed), mean ages of smokers and non-smokers, type of exposure to smoking (active cigarette smoking or second-hand smoker), frequency of poor outcomes among smokers, frequency of successful outcomes among smokers, frequency of poor outcome among non-smokers, frequency of successful outcomes among non-smokers, and measure of effect used by the study and the corresponding p-value (S2 Annex). In cases of incomplete data, we contacted authors. However, none could provide additional information stating that data were old and could not be retrieved easily from archives. For this review, ex-smokers or previous-smokers that stopped smoking six weeks or more before initiation of TB treatment were categorized as non-smokers with respect to immune response [6, 7].

### Methodological quality assessment

We assessed the quality of non-randomized studies using the Newcastle Ottawa Quality Assessment Scale [20]. This scale assigns a “star” based on three broad perspectives: the selection of the study groups; the comparability of the groups; and the exposure for case-control or cross-sectional studies, or the outcome for cohort studies. The maximum possible score awarded to a study was 9 points. A score of <5 was considered low quality. A score of 5 to 7 was considered medium quality, whereas >7 was considered high quality. Subsequently, we carefully reviewed the influence of studies rated with less than 5 stars on summary effect size. For randomized clinical trials, we used the Cochrane Collaboration’s tool for assessing the risk of bias [21] to assess methodological quality. For non-randomized quasi-experimental studies, we used the Joanna Briggs Institute (JBI) Critical Appraisal Checklist to assess the risk of bias [22].

### Data analysis

We executed most of the statistical analyses using the meta package add-on in Stata software version 14. We used Comprehensive Meta-Analysis (CMA) version 3 software for other analyses, such as Orwin’s Fail-Safe N, because they cannot be run on Stata.

We applied the random-effects model for the quantitative analysis and generated odds ratios (ORs) for individual studies with their 95% confidence intervals, and visualized summary odds ratio using a forest-plot. We assessed the heterogeneity of the studies using I-squared and the p-value for Q-statistic. We also performed a sensitivity analysis to test whether there was a substantial difference in the conclusion reached on the summary effect size and determine how robust the summary odds ratios were by omitting each study one at a time.

We classified countries where the studies were conducted according to categories of low- and middle-income, upper-middle-income and high-income and performed subgroup analyses by income strata, the period of the studies as before or in 2010, and after 2010 and HIV infection status. Reasons for heterogeneity were further explored applying meta-regression analysis on the year of studies adjusting for income level.

### Publication bias

We investigated publication bias by using a funnel plot which is inherently subjective, Egger’s test of significance of bias and contour-enhanced funnel plot, successively. Additionally, we conducted a trim and fill analysis which iterates computation of estimate effect size until symmetry of funnel plot is reached to check for the effect of publication bias if any trimmed study is shifting the unadjusted summary effect size. Finally, we checked for a small study effect by running a cumulative meta-analysis which also serves as a kind of sensitivity analysis. We also ran Orwin’s Fail-Safe N.

## Results

**Fig 1:**
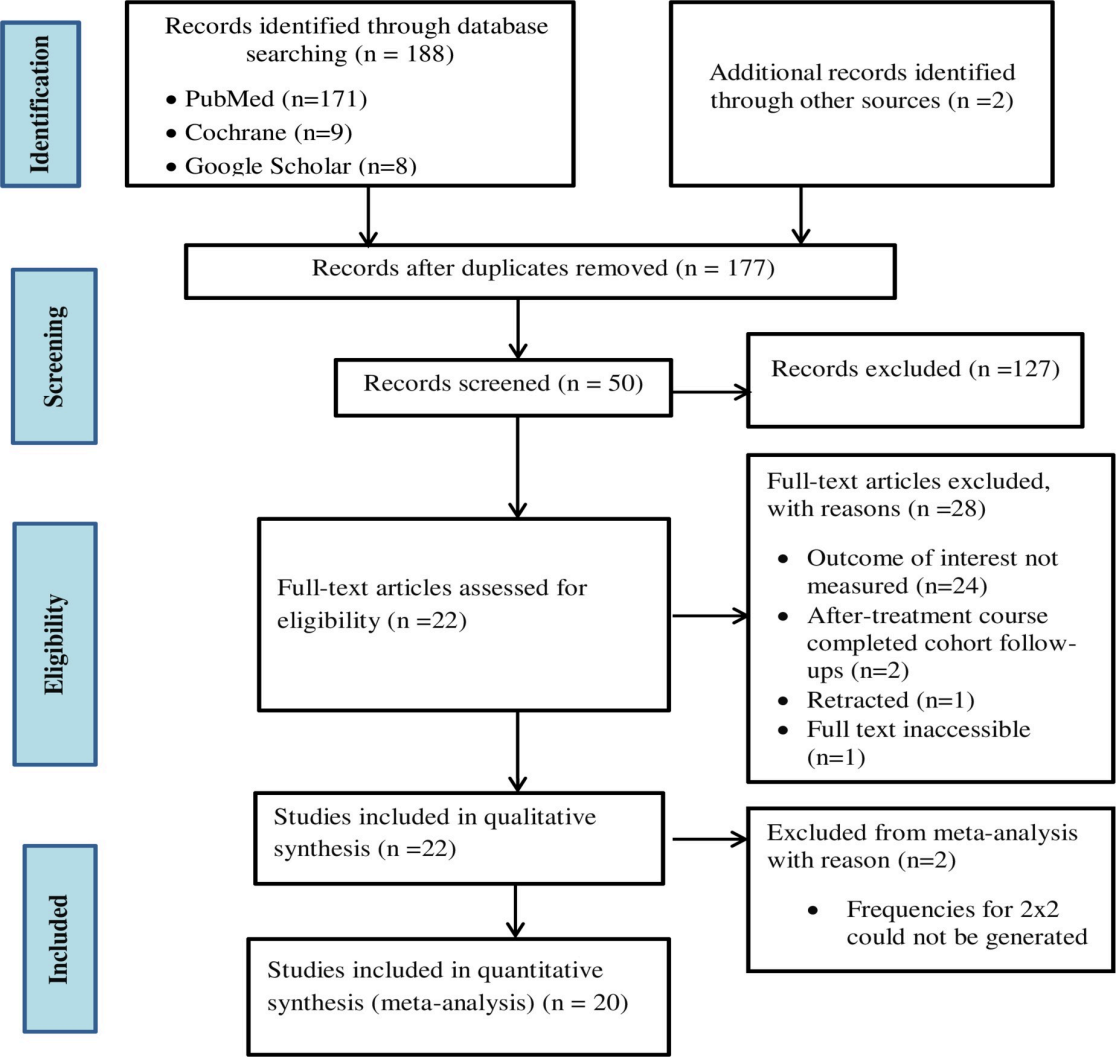
Flow chart for selecting studies for the systematic review and meta-analysis.

We used the Preferred Reporting Items for Systematic Reviews and Meta-analyses (PRISMA) guidelines (S1 Text) [23] to conduct this systematic review and meta-analysis. We retrieved a total of 190 records (171 from PubMed/MEDLINE, 9 from the Cochrane library and 8 from Google Scholar) on or before February 27, 2019. After removing duplicates, 177 remained for further evaluation. Screening titles and abstracts eliminated 127 records because of the following reasons; their topics were unrelated, article comprised of guidelines, position paper, review or only a title without and abstract. Out of the remaining 50 articles screened, 22 met the eligibility criteria and were included in the qualitative synthesis. We excluded 29 articles from the analysis because 18 did not measure TB treatment outcomes, 6 only measured outcomes before treatment course completed (checking sputum culture conversion at 2 months following initiation of the treatment but comparing the outcomes among smokers and non-smokers), 2 were extended follow-up studies after the initial follow-up completed, 1 article was watermarked as retracted and we could not access the full text for 1 article. In the meta-analysis, we excluded a further two articles [24, 25] among those 22 articles eligible for qualitative synthesis because the articles did not report frequencies for cells of 2x2 tables and we could not compute them from summary statistics reported in the articles. See Fig 1 for the study selection flow chart according to PRISMA guidelines.

### Risk of bias assessment

The overall scores for the risk of bias assessment of observational studies according to the Newcastle-Ottawa scale were 7 and greater except for two cross-sectional studies; one conducted in Iran [26] and the other in Fiji [27] that scored 4 and 5, respectively. The clinical trial [28] and quasi-experimental [29] studies were assessed based on Cochrane collaboration quality assessment and JBI checklists, respectively (S1 Table).

### Characteristics of the included studies

We pinpointed twenty-two studies eligible for the qualitative synthesis. The studies were conducted between 1999 and 2014, except the study by Reed, et al which did not mention dates but whose publication was in 2013. The most recent studies were published in 2019. Study designs included: 10 cohort studies [9, 24, 25, 30–36], 8 cross-sectional studies [26, 27, 37–42], 2 case-control studies [43, 44], 1 clinical trial [28] and 1 quasi-experimental study [29]. Except for one study [27] which did not specify the type of exposure (active or passive) to tobacco/cigarette smoking, the remaining 21 studies compared TB treatment outcomes among individuals exposed to active cigarette smoking versus non-exposed. Twelve studies reported on adult participants of 18 years and above [9, 24, 26, 28, 32–35, 38, 40–42]. Eight studies failed to mention whether they included any participants under 18 [25, 29–31, 36, 39, 43, 44]. Only two studies specifically involved children below 18 years of age as study participants [27, 37], with 6% of participants for the first cited and 1.5% for the second study. We also scrutinized the magnitude of HIV infection in the studies. We could not find HIV data in the report of 6 of the 22 studies that qualified for the qualitative synthesis [25, 27, 35, 38, 41, 44]. Five studies restricted their assessments of the effect of smoking on TB treatment outcomes to HIV uninfected participants only [24, 28, 29, 34, 43]. The proportion of study participants living with HIV was reported in 11 studies [9, 26, 30–33, 36, 37, 39, 40, 42].

We also extracted data on whether the TB patients had any drug susceptibility in the individual studies. TB drug susceptibility among study participants was not stated in 10 studies [25–27, 33, 36, 38–40, 42, 44]. Five studies [28, 29, 31, 35, 43] assessed treatment outcomes in patients who were susceptible to first-line TB treatment and 2 studies [32, 34] evaluated treatment outcomes in association with smoking among multi-drug resistant (MDR) TB patients. The remaining 5 studies reported on a mix of drug-susceptible and MDR-type TB participants [9, 24, 30, 37, 41]. The types of TB treatment outcomes reported varied among studies. Twelve studies assessed all the three poor outcomes, i.e., died, treatment failure and loss to follow up (LTFU) [9, 26–28, 30, 31, 34, 35, 39–41, 43]. The measures of association that studies used to analyze the effect of smoking on TB treatment outcomes included adjusted odds ratio (aOR) [25, 30, 31, 33, 37, 42–44], crude odds ratio (COR) [27, 28, 36, 39, 40], chi-square test [29, 34, 38], adjusted risk ratio (aRR) or adjusted hazard ratio (aHR) [9, 24, 32, 41], and proportion [26, 35].

Overall, 12 studies reported p-values (<0.05) which implied that smoking significantly increases risk of poor tuberculosis treatment outcomes [9, 25, 28–31, 33, 35, 37, 40, 42, 43]. However, three studies indicated that the effect of smoking was dependent on the dose of the cigarette smoked per day [30, 42, 43]. The studies that reported the dose-dependent effect used a variable number of cigarettes smoked per day to compute the effect of smoking as compared with non-smokers. One of these studies used 11 or more cigarettes as cut off number of cigarettes smoked per day and reported P-value of 0.026 [30], the other used more than 20 cigarettes per day and reported P-value of 0.047 [43] and the last one used more than 50 packs per year (roughly two or more cigarettes per day) and reported a P-value of 0.028 [42].

Conversely, ten studies reported non-significant p-value (>0.05) for the effect smoking on TB treatment outcomes [24, 26, 27, 32, 34, 36, 38, 39, 41, 44]. One study reported no effect irrespective of the number of cigarettes smoked per day, with a P-value of 0.29 for less than one pack (12 cigarettes) per day smokers and 0.14 for one or more pack per day [24]. The study by Tabarsi et al. [26] which compared the proportion of outcomes among smokers and non-smokers reported a p-value of > 0.05.

Among the ten observational studies that reported that smoking was linked with poor treatment outcomes, seven studies [9, 25, 30, 31, 37, 40, 42] had a quality assessment score of 9 stars, two studies [33, 43] scored 8 and one study [35] scored 7 out of scale of 9 based on the New Castle-Ottawa tool as shown in S1 Table. We rated the other two studies, a clinical trial [28] and a quasi-experimental [29] study that concluded that smoking cessation protects against poor treatment outcomes as having a low risk of bias. Six of these studies were cohort studies [9, 25, 30, 31, 35], three were cross-sectional [37, 40, 42], two were experimental [28, 29] and one was a case-control [43].

Among 12 studies that concluded that smoking is linked with poor treatment outcomes, eight studies [9, 25, 30, 31, 33, 37, 42, 43] computed adjusted measure of association and four studies [28, 29, 35, 40] computed crude statistic. Among 8 studies that reported adjusted effect sizes, seven studies [9, 25, 30, 33, 37, 42, 43] adjusted for both sex and age. Three studies [30, 31, 37] also adjusted for HIV infection along with other variables like sex, age, MDR TB and BCG scar in Liew et al. [37], sex, TB re-treatment and overcrowding in Leung et al. [31], and sex, age, residence, TB drug resistance, overcrowding and hemoptysis in Bonacci et al. [30]. In addition to sex and age, the effects of two studies [42, 43] were adjusted for both body mass index and diabetes Mellitus.

Among 12 studies reporting HIV infection status, all participants in three studies were HIV uninfected [28, 29, 43], varying proportions of participants were living with HIV in six studies [9, 30, 31, 37, 40, 42]. HIV infection was not specified in two studies [25, 35], and all participants were living with HIV in 1 study [33].

Among the 12 studies where smoking was linked with poor outcomes, in five of them [28, 29, 31, 35, 43] study participants were exclusively susceptible to first-line TB drugs. Four studies did not specify whether participants had drug-resistant or drug-susceptible TB or a mix of both [25, 33, 40, 42], whereas a mix of participants was reported in 3 studies [9, 30, 37].

All 10 studies that failed to show a significant association between smoking and poor TB treatment outcomes were observational studies. Five were cross-sectional/record review [26, 27, 38, 39, 41], four were cohort [24, 32, 34, 36] and one was a case-control study [44].

The quality score of studies, indicated in Table 1, was the maximum 9 score for three studies [24, 41, 44], 8 for two studies [32, 39], 7 for three studies [34, 36, 38], and score of 5 for one study [27] and 4 for the other one study [26].

**Table 1:** Characteristics of studies included in the review and values of their effect measures with corresponding p-values.

First author	Study year	Study country	Study design	Measured Poor outcome	Proportion of HIV infected participants	TB drug Susceptibility status of participants	Effect measured by	p-value reported	Sample size
Leiw, et al [37]	2012	Malaysia	Cross-sectional	Died, Failure, LTFU, and TO	6.6%	Mixed participants	aOR	0.011	21426
Leung, et al [31]	2001–2003	Hong Kong	Cohort	Died, Failure, and LTFU	0.43%	Susceptible	aOR	0.001	15891
Salami, et al [40]	1991–1999	Nigeria	Cross-sectional	Died, Failure, and LTFU	4.25%	Not specified	COR	0.001	1530
Magee, et al [32]	2009–2012	Georgia	Cohort	Died, Failure, LTFU, and TO	4.5%	MDR only	aRR	>0.05	1321
Przybylski, et al [39]	2001–2010	Poland	Cross-sectional	Died, Failure, and LTFU	0.4%	Not specified	COR	0.930	1997
Bonacci, et al [30]	1995–2010	Mexico	Cohort	Died, Failure, and LTFU	2%	Mixed participants	aOR	0.026 or 0.2*	1022
Yamana, et al [42]	2010–2013	Japan	Cross-sectional	Died	0.1%	Not specified	aOR	0.028 or 0.123**	762
Wang, et al [41]	2002–2003	Taiwan	Cross-sectional	Died, Failure, and LTFU	Not specified	Mixed participants	aHR	>0.05	523
Gegia, et al [9]	2011–2013	Georgia	Cohort	Died, Failure, and LTFU	1.35%	Mixed participants	aRR	<0.050	524
Chiang, et al [43]	2001–2003	Taiwan	Case-control	Died, Failure, and LTFU	0%	Susceptible	aOR	0.047 or 0.073***	302
Maruza, et al [33]	2007–2009	Brazil	Cohort	LTFU	100%	Not specified	aOR	0.007	273
Masjedi, et al [28]	2012–2014	Iran	Clinical trial	Died, Failure, and LTFU	0%	Susceptible	COR	0.001 or 0.07****	334
Roy, et al [44]	2011	India	Case-control	LTFU	Not specified	Not specified	aOR	0.720	158
Alo, et al [27]	2010–2012	Fiji Island	Cross-sectional	Died, Failure, and LTFU	Not specified	Not specified	COR	0.500	375
Ma, et al [38]	2008–2011	China	Cross-sectional	Died and Failure	Not specified	Not specified	chi2-test	0.076	791
Silva, et al [36]	2005–2007	Brazil	Cohort	Died	70.8%	Not specified	COR	0.570	140
Pazarli, et al [34]	2000–2005	Turkey	Cohort	Died, Failure, and LTFU	0%	MDR only	Chi2-test	0.190	103
Rathee, et al [35]	2010–2011	India	Cohort	Died, Failure, and LTFU	Not specified	Susceptible	Proportion	0.000	101
Awaisu, et al [29]	2008–2009	Malaysia	Quasi-experimental	LTFU and Failure	0%	Susceptible	Chi2-test	0.043	86
Tabarsi, et al [26]	2004–2007	Iran	Cross-sectional	Died, Failure, and LTFU	100%	Not specified	Proportion	>0.05	111
Reed, et al [24] ^π^	Not stated^Ψ^	Republic of Korea	Cohort	Died	0%	Mixed participants	aHR	0.29 or 0.14*****	657
Tachfouti, et al [25] ^π^	2004–2009	Morocco	Cohort	Failure	Not specified	Not specified	aOR	0.030	727

aOR = adjusted odds ratio, COR = Crude odds ratio, aRR = adjusted risk ratio

aHR = adjusted hazard ratio, LTFU = Loss to follow-up, TO = transferred out

^Ψ^published in 2013

^π^Not eligible for the meta-analysis

*p-value of 0.026 for heavy (≥11cigarette/day) & 0.2 for light smokers (<11 cigarette/day)

**p-value of 0.028 for >50packs/year smokers and 0.123 for ≤ 50packs/year smoker

***p-value of 0.047 for >20 cigarette/day smokers and 0.073 for 1-20/day

****p-value of 0.001 for smoker and 0.07 for quitters at treatment initiation

***** p-value of 0.29 for <1 pack/day smokers and 0.14 for ≥ 1 pack/day smokers

Among these 10 studies, six studies reported unadjusted measures of association between smoking and poor TB treatment outcomes [26, 27, 34, 36, 38, 39]. Tabarsi et al [26] reported an effect of smoking with a p-value > 0.05 but without showing the effect size. Measures of association were adjusted for both age and sex in the remaining four studies [24, 32, 41, 44]. In addition to controlling for age and sex covariates, Magee et al [32] controlled for HIV infection, body mass index and diabetic Mellitus, whereas Wang et al controlled for multi-drug resistance [41].

We assessed the type of study participants with regard to TB drug susceptibility and we found that six studies [26, 27, 36, 38, 39, 44] out of the 10 studies did not specify the type of their study participants. Two studies [32, 34] were conducted on drug-resistant TB patients and two other studies [24, 41] were conducted on participants who were a mix of TB drug-susceptible and resistant.

Characteristics of the study participants of the studies that reported an insignificant association between smoking and TB treatment outcomes with regard to HIV infection status were observed as; 3 studies reported proportion on infected participants as 4.5% [32], 70.8% [36] and 0.4% [39]. One study was conducted on exclusively HIV infected participants [26] and participants of 2 studies were HIV uninfected population [24, 34]. HIV infection proportion was not clearly indicated in 1 study [41] though it clued there were infected participants and 3 studies didn’t clearly describe the HIV infection status of their study participants [27, 38, 44]. Table 1 has summarized characteristics of the studies and measures of effect with their p-values. The table does not include data extracted on whether the study included participants under 18 years old and other adjusted variables stated above.

### Association of smoking with TB treatment outcomes

**Fig 2:**
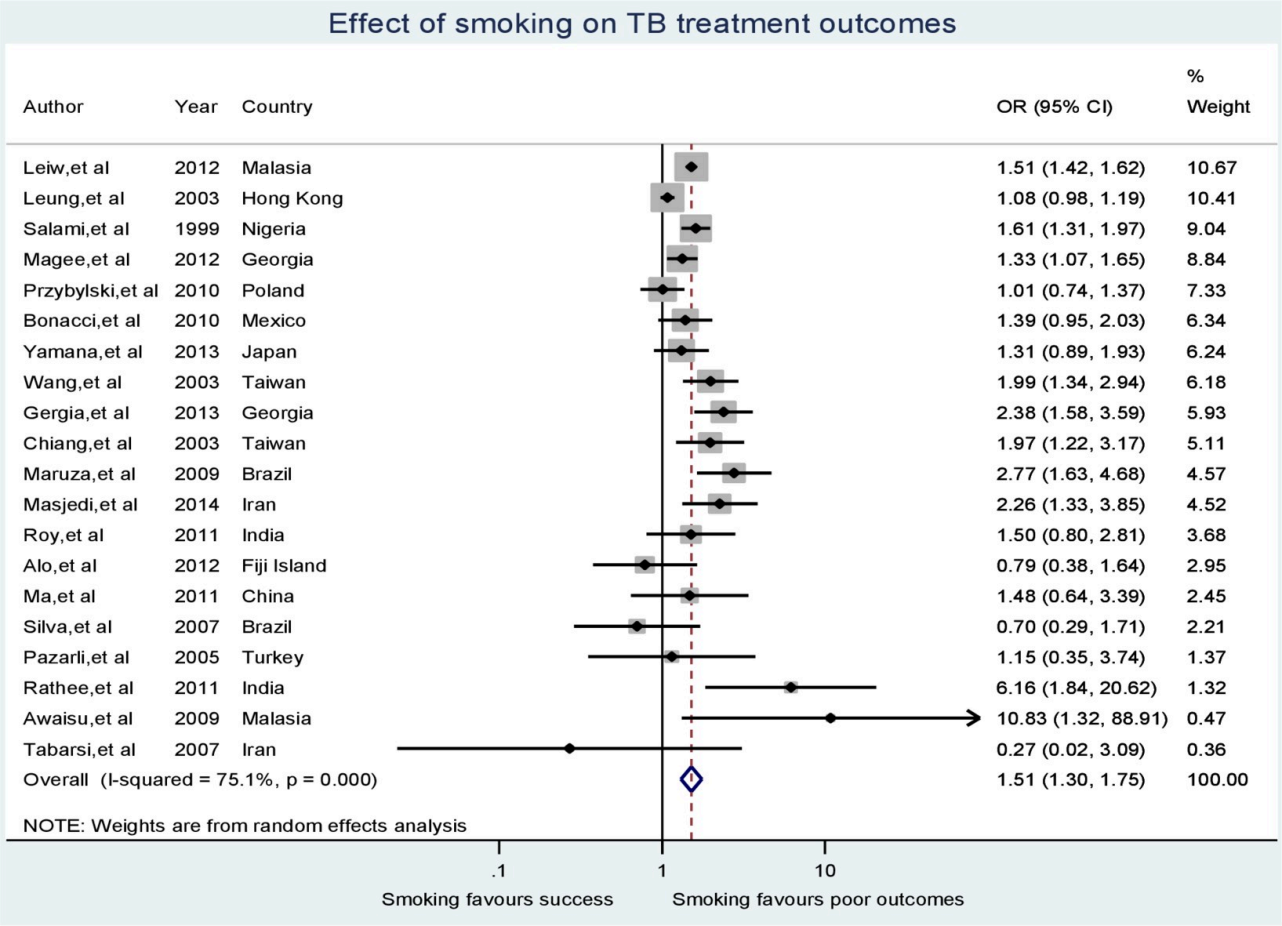
A forest plot displaying the effect of smoking on TB treatment outcomes.

Sample sizes of the studies included the meta-analysis ranged from 86 [29] to 21,426 in the largest study [37]. Across all studies, there were 47,770 participants. Smokers accounted for 33% of total participants. TB treatment outcomes were poor for 21% of all study participants. Patients with poor TB treatment outcomes were 50% more exposed to smoking than patients with successful treatment (OR 1.51, 95% CI 1.30–1.75). Heterogeneity was measured using I-squared indicating that about 75% of the observed variability among study odds ratios was attributed to true heterogeneity between the studies (I-squared = 75.1%, p < 0.001). Fig 2 demonstrates the odds ratios of individual studies, summary odds ratio and test of heterogeneity employing a forest plot.

### Sensitivity analysis

**Fig 3:**
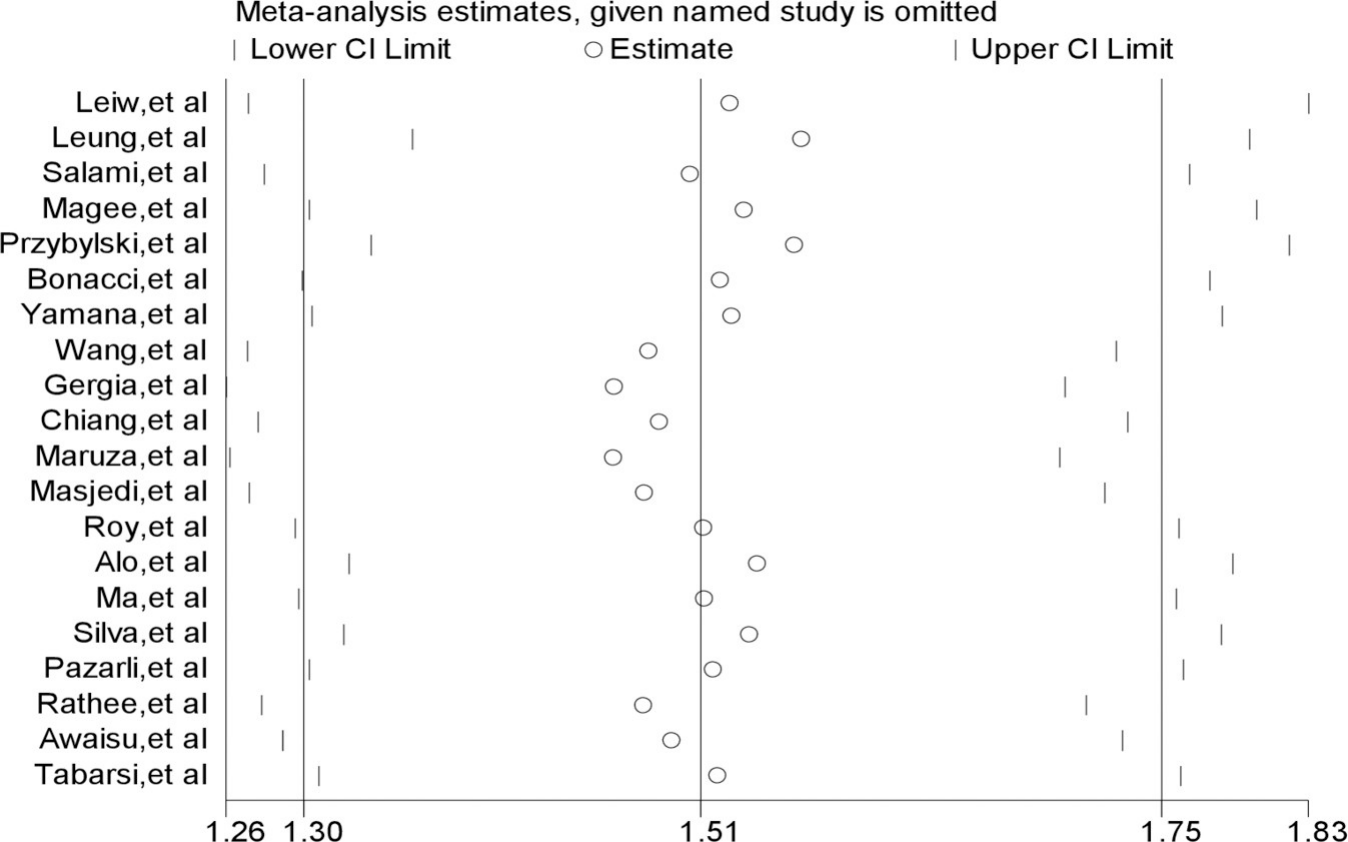
Influential analysis for studies included in the meta-analysis.

We performed a sensitivity analysis to investigate the influence of each individual study on the overall summary odds ratio by omitting each study turn by turn and re-estimating the summary odds ratio. As seen in Fig 3, there was no single study for which the point estimate of its omitted analysis (the small circle) lies outside of the confidence interval of the combined meta-analysis represented by the solid vertical lines. Rather, point estimates of each omitted analysis within the entire set of studies cluster around the point estimate of the combined meta-analysis (the middle solid vertical line).

### Sub-group analysis

**Fig 4:**
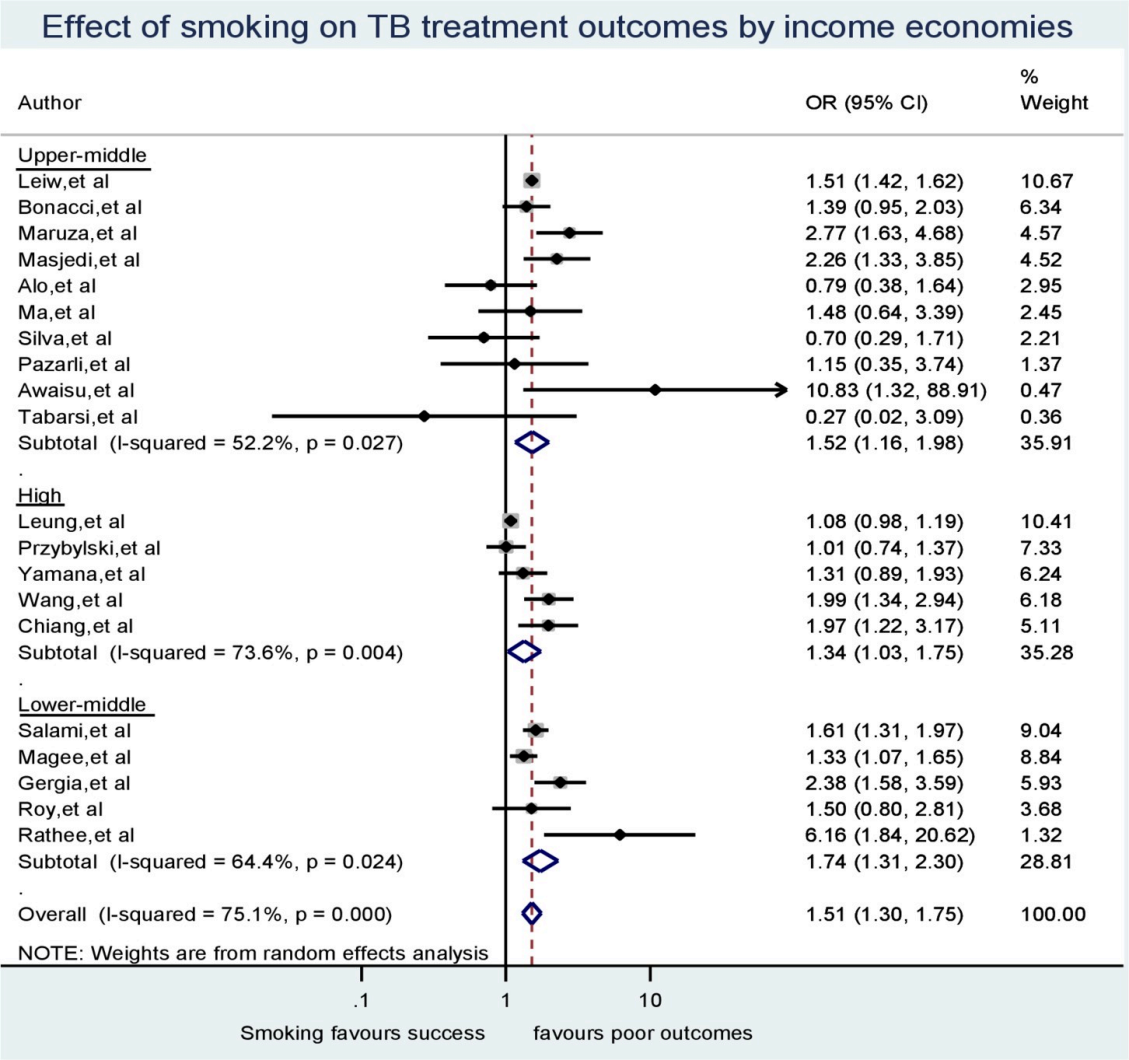
Subgroup meta-analysis for the effect of smoking on TB treatment outcomes by income economies.

Our first subgroup analysis, as to whether the context of study with respect to the type of economy in the country where the study took place, found that smoking was significantly associated with poor tuberculosis outcomes irrespective of the income category of the study country’s economy. The odds ratios were 1.74 (95% CI = 1.31–2.30), 1.52 (95% CI = 1.16–1.96) and 1.34 (95% CI = 1.03–1.74) for lower-middle-, upper-middle- and high-income economies, respectively. In the sub-group analysis, we found that heterogeneity between studies’ odds ratios for lower-middle, upper-middle and high-income countries decreased from the crude I-squared of 75.1% (p < 0.001) to I-squared values equal to 64.4% (p = 0.024), 52.2% (p = 0.027) and 73.6% (p = 0.004), respectively as indicated in Fig 4. No study in our analysis came from a lower-income country.

**Fig 5:**
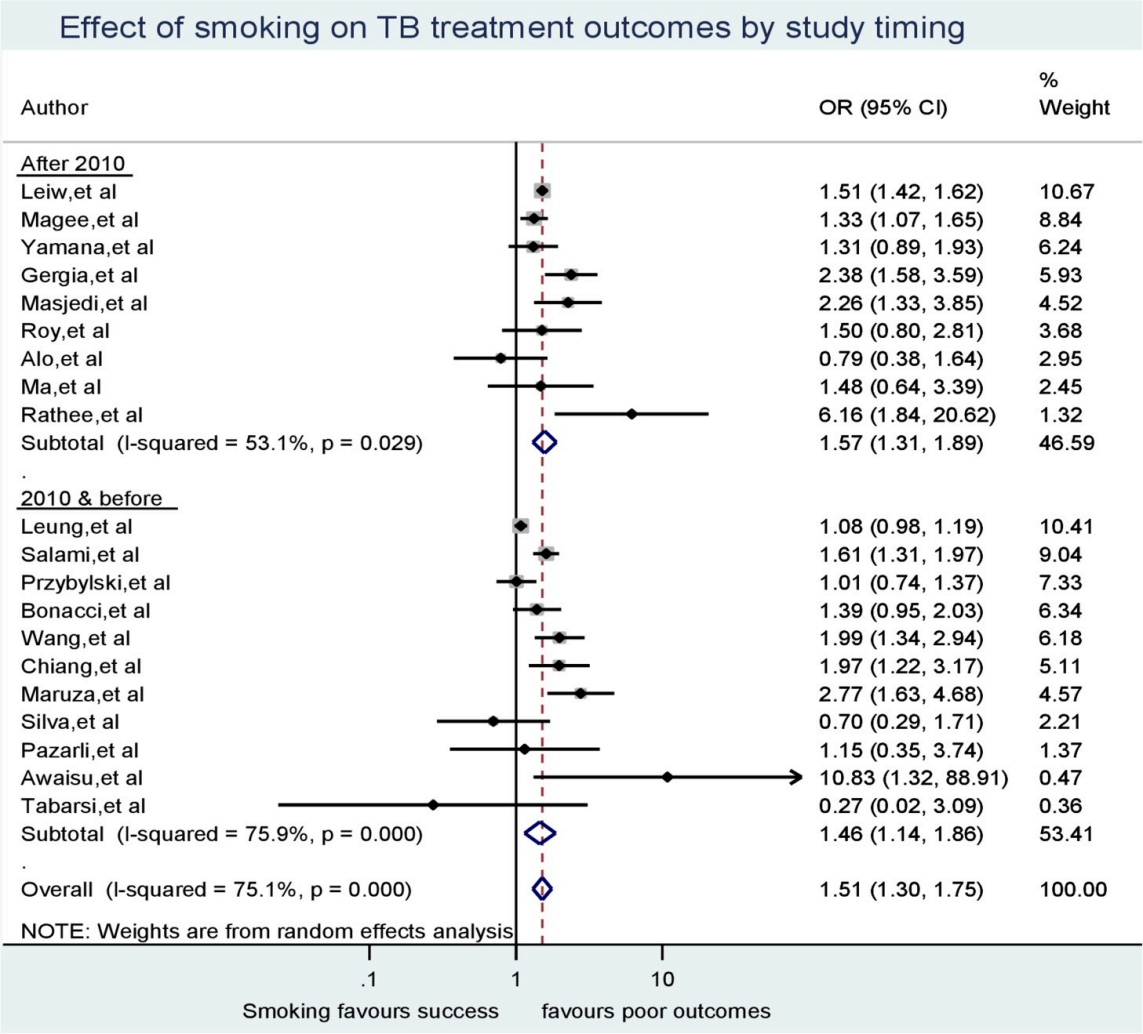
Sub-group analysis for the effect of smoking on TB treatment outcomes according to study timing.

Our second subgroup analysis related to whether a study was conducted before or during 2010, or thereafter found that studies conducted during 2010 and before demonstrated high between-study heterogeneity (I-squared = 75.9%, p < 0.001), even though the effect of smoking on TB treatment outcomes favored poor outcomes in both periods. Conversely, the variability of odds ratios between studies conducted after 2010 was smaller (I-square = 53.1%, p = 0.029) (Fig 5).

**Fig 6:**
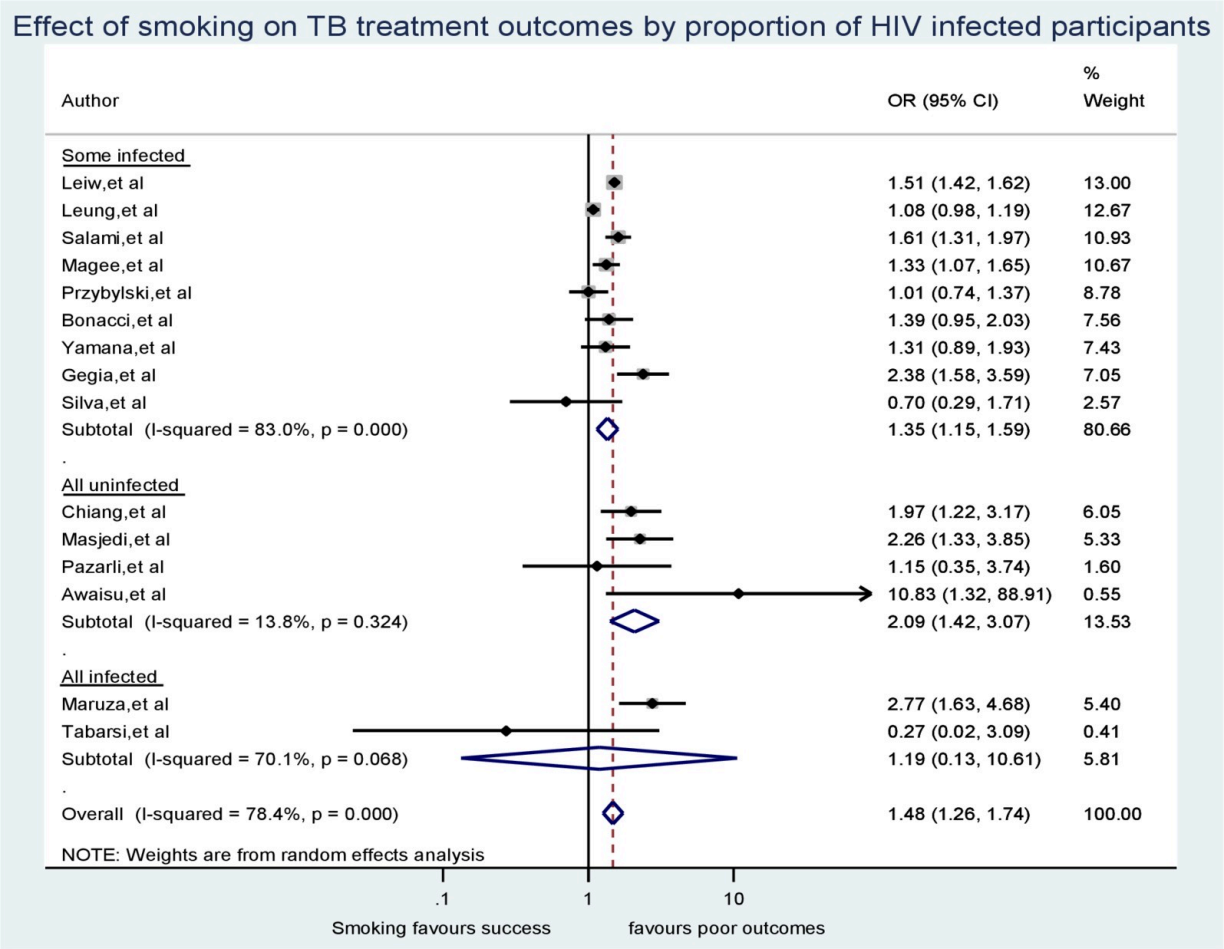
Sub-group analysis according to the proportion of study participants living with HIV.

To further investigate the source of heterogeneity, we ran a subgroup analysis by HIV status in study participants of individual studies. While doing so, we excluded the 5 studies with no HIV status reported for their participants [27, 35, 38, 41, 44]. We display the output of this subgroup analysis (Fig 6). From the subgroup analysis, we found that there was insignificant heterogeneity between studies whose participants were not affected by HIV (I-squared = 13.8%, p = 0.324). However, heterogeneity remained significant between studies conducted on study participants with mix in HIV status (I-squared = 83.0%, p < 0.001), and between those studies carried out among only people living with HIV (I-squared = 70.1%, p < 0.001).

### Meta-regression

The timing of when the study was undertaken and the study country’s income category were fit to a meta-regression model to investigate whether these study-level covariates had driven the heterogeneity. Lower-middle income was used as a reference category in the regression analysis. The joint test for both covariates gave a p-value of 0.78, indicating no association for at least one of the covariates. More than 64% (I-squared residual = 64.63%) of the observed variation in odds ratios of the effect of smoking among the studies was attributed to between-study variations. The negative adjusted R-squared (-37.04%) implies the covariates explain less of the heterogeneity than would be expected even by chance alone (S2 Table).

## Publication bias

**Fig 7:**
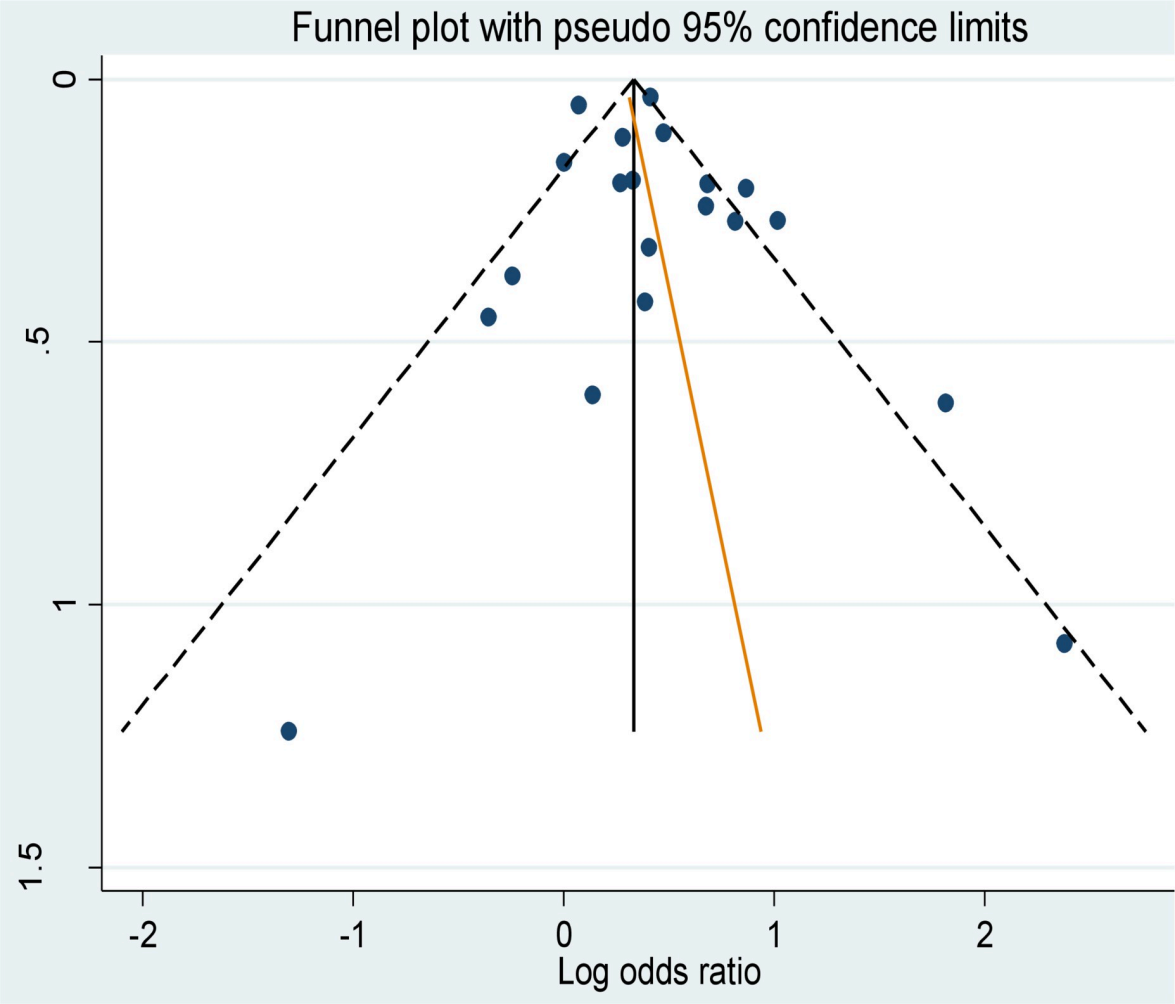
Funnel plot of the studies of the effect of smoking on TB treatment outcomes.

According to the funnel plot shown in Fig 7, the studies appear visually to be distributed symmetrically about the mean effect size represented by the solid vertical line. This indicates that publication bias was unlikely for the computed effect of smoking on poor treatment outcomes. The oblique line that is shown superimposed on the funnel plot is Egger’s regression line.

We performed the Egger’s statistical test of symmetry and found that there was no statistically significant association between studies’ effect sizes and their standard errors as the p-value is insignificant (Egger’s bias coefficient = 0.52, p = 0.403).

**Fig 8:**
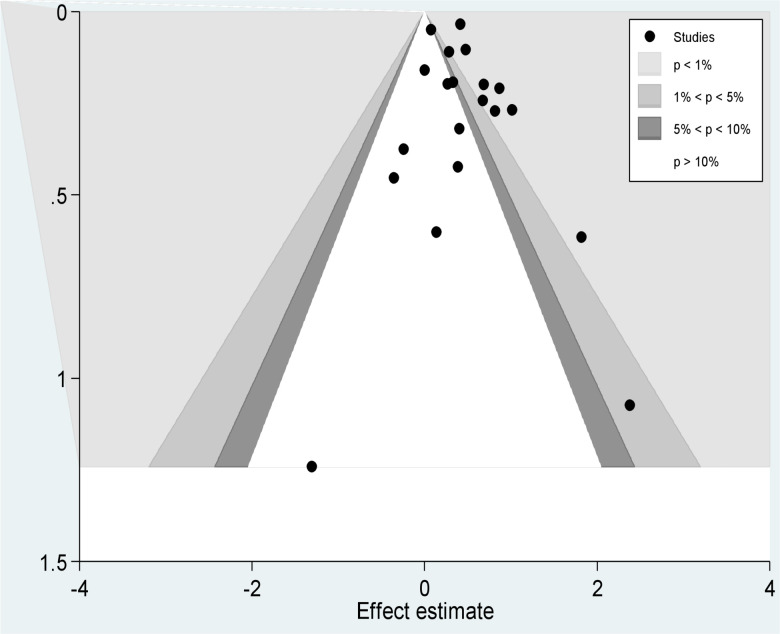
Contour enhanced funnel plot of studies of effect of smoking on TB treatment outcomes.

We also applied a contour enhanced funnel plot to investigate the area where the missing studies are perceived. As sees in Fig 8, small studies and large studies that reported smoking significantly decreased the likelihood of poor tuberculosis treatment outcomes were missing on the contours of the statistical significant area on the left side of the plot. Nevertheless, small studies that were reporting no statistically significant association were not missing.

Furthermore, we evaluated the robustness of the combined odds ratio using the trim and fill analysis and found an unbiased/adjusted odds ratio from the random-effects model of 1.47 (95% CI = 1.26 to 1.71) and an unadjusted/observed one of 1.51 (95%CI = 1.30 to 1.75) (Table 2).

**Table 2:** Duval and Tweedie’s trim and fill for the effect of smoking on tuberculosis treatment outcomes.

	Fixed Effect				Random Effects		
	Studies Trimmed	Point Estimate	Lower Limit	Upper Limit	Point Estimate	Lower Limit	Upper Limit
**Observed values**		1.396	1.330	1.465	1.511	1.303	1.751
**Adjusted values**	2	1.391	1.326	1.460	1.467	1.260	1.708

Using Orwin’s Fail-Safe N method, we determined that it would take 50 missing studies with a mean odds ratio of 1.0 to bring the overall effect of 1.51 to a value we selected of an effect of 1.10 and become trivial. The classic (Rosenthal’s) fail-safe N, which computes the number of hidden studies required to make the effect not statistically significant was 536 (S3 Table).

### Cumulative meta-analysis

**Fig 9:**
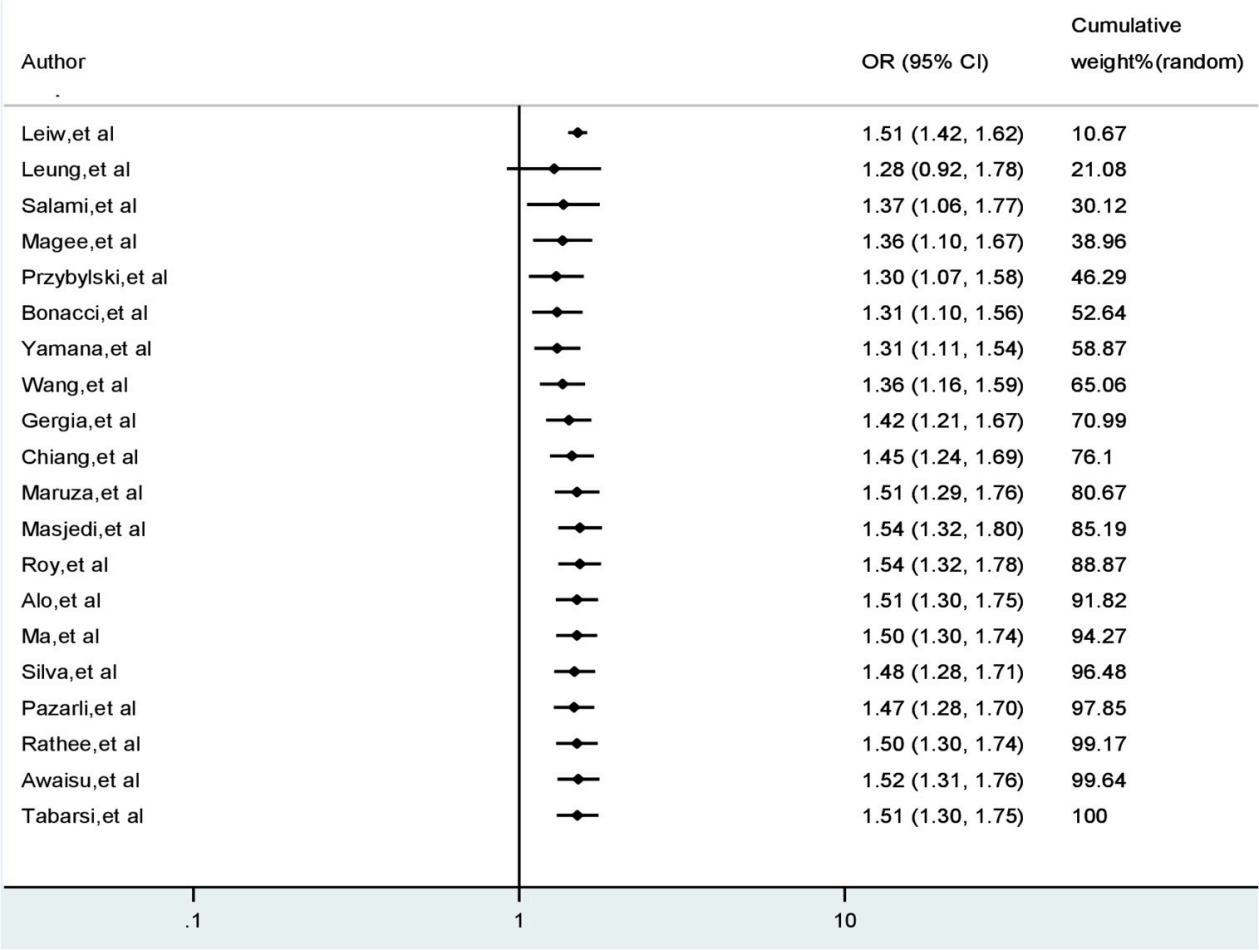
Cumulative meta-analysis of the effect of smoking on TB treatment outcomes.

In order to restrict the analysis to larger studies only, we sorted the 20 studies from most precise to least precise which roughly correspond to sorting from largest to smallest sample size. With the 11 largest studies in the analysis, the cumulative odds ratio became 1.51. With the addition of the remaining 9 smaller studies, the point estimate did not consistently shift to any side, and the odds ratio remained 1.51. The fact that the analysis incorporating all 20 studies assigns 80.7% of its weight to the 11 larger studies, as shown in the right column of Fig 9, implies that smaller studies contributed less weight and thus cannot introduce bias.

## Discussion

This systematic review and meta-analysis aimed to evaluate the association between smoking and TB treatment outcomes. We identified no study that reported smoking significantly favors successful TB treatment outcomes. Instead our pooled estimate of odds ratios found that active cigarette smoking is significantly associated with poor TB treatment outcomes. High heterogeneity suggests we should be cautious in generalizing the pooled mean odds ratio estimate to different populations [45]. This considerable statistical heterogeneity in odds ratios between studies could arise from methodological or clinical diversity or, in all likelihood, a combination of both. The review included studies with diverse designs including experimental, cohort, case-control and cross-sectional studies [46]. Clinical diversity is also likely because studies included were diverse in their participants’ characteristics (HIV status, TB drug susceptibility), the TB treatment outcomes measured, study context (country income category) and study periods [47].

Contrarily, a meta-analysis by Samuels JP et al showed that there is no difference between smokers and non-smokers. However, the fact that their analysis was based on studies involving only MDR/XDRTB patients may be the reason for our differences [48].

In this meta-analysis, pooling changed the significance of effects originally found in individual studies from significant to not significant and vice versa. The studies by Bonnaci et al [30] and Yamana et al [42] were reporting significant p-values but their effects were no longer significant in the meta-analysis. The loss of significance resulted from aggregating light smokers and heavy smokers together as smokers instead of comparing them separately with non-smokers in the original studies. Similarly, ex-smokers and current smokers were independently compared with never-smokers in Leung et al [31] but ex-smokers (“an ever smoker who had stopped smoking for at least for 1 year before the current TB episode” [31]) were merged with non-smokers in this meta-analysis under the assumption that 1 year offers sufficient recovery time from the effect of smoking [6, 7]. On the other hand, Magee et al’s originally non-significant p-value for adjusted effect size shifted to significance in the meta-analysis (as defined by confidence intervals that do not contain the null value OR of 1). This could be explained by a larger sample size (i.e. crude frequency that is not unadjusted for different factors) and smaller p-value (tendency to be significant) effect [49].

We ran a sensitivity analysis to investigate the influence of each individual study on the overall summary estimate that found that omitting any of the studies had no excessive influence on the summary odds ratio [50].

We investigated sources of heterogeneity based first on country income category [51]. Doing so reduced the level of heterogeneity to moderate level [45] implying that some variability between studies was attributable to context even if not statistically significant. Second, we explored whether timing of study implementation before or during 2010 or after 2010 explained any variability between studies and found less variability in later than earlier studies. We also concluded that diversity in HIV status among study participants was responsible for heterogeneity in the effect of smoking where studies conducted on participants not living with HIV had insignificant heterogeneity. We explain the significant heterogeneity in studies involving people living with HIV on the variability in immunity levels in these populations depending on clinical stages of their disease [52].

Finally, by adjusting for country income category and timing of the studies, our meta-regression analysis sought to assess whether the effect of smoking decreased over time in conjunction with progressive reductions in nicotine content of cigarettes [53]. However, we learned that the effect of smoking on TB treatment outcomes neither significantly changed overtime nor showed difference among study countries’ income category. Therefore, it was unlikely that the year of study or the income category of studies’ countries caused the heterogeneity. Our analyses also indicate that publication bias was unlikely.

## Limitations

This review has relied entirely on searching free electronic study databases. Thus it is likely we missed studies indexed in health sciences databases like Embase. Using odds ratios as the measure for pooling of effect sizes where the magnitude of poor TB treatment outcome was not a rare event (21%) and approximating a risk ratio is another limitation of this review. The search language restricted to English may have led to missing additional work published in other languages.

## Conclusion

Smoking is significantly linked with poor tuberculosis treatment outcomes, particularly in lower-middle-income and upper-middle-income countries as compared to high-income countries though the difference was not statistically significant.

## Supporting information

S1 Annex Summary of output from databases searches for a systematic review and meta-analysis on the effect of smoking on TB treatment outcomes. (DOCX)

S2 Annex Data-extraction sheet for a systematic review and meta-analysis on the effect of smoking on TB treatment outcomes. (XLSX)

S1 Table Risk of bias assessment of the included studies using the Newcastle-Ottawa quality assessment scale. (DOCX)

S2 Table Meta-regression of the study timing and income category on the effect of smoking on tuberculosis treatment outcomes. (DOCX)

S3 Table Orwin’s Fail-Safe N analysis for the effect of smoking on poor tuberculosis treatment outcomes. (DOCX)

S1 Text PRISMA checklist for a systematic review and meta-analysis on the effect of smoking on TB treatment outcomes. (DOC)
