# Supplementary material for: Effect of smoking on tuberculosis treatment outcomes: A systematic review and meta-analysis
Source: PLoS One. 2020 Sep 17;15(9):e0239333. doi: 10.1371/journal.pone.0239333 (PMC7498109; doi:10.1371/journal.pone.0239333)
Supplement: S1 Annex — (DOCX) [file pone.0239333.s001.docx]

# S1 Annex. Summary of output from databases searches **for a systematic review and meta-analysis on the effect of** smoking on TB treatment outcomes

**PubMed advanced searching search terms used and the downloaded history**

**FROM Search detail box**: (("tuberculosis"[MeSH Terms] OR "tuberculosis"[All Fields]) AND ("treatment outcome"[MeSH Terms] OR ("treatment"[All Fields] AND "outcome"[All Fields]) OR "treatment outcome"[All Fields])) AND (((("smoking"[MeSH Terms] OR "smoking"[All Fields]) OR ("smoking cessation"[MeSH Terms] OR ("smoking"[All Fields] AND "cessation"[All Fields]) OR "smoking cessation"[All Fields])) OR ("cigarette smoking"[MeSH Terms] OR ("cigarette"[All Fields] AND "smoking"[All Fields]) OR "cigarette smoking"[All Fields])) OR ("tobacco smoke pollution"[MeSH Terms] OR ("tobacco"[All Fields] AND "smoke"[All Fields] AND "pollution"[All Fields]) OR "tobacco smoke pollution"[All Fields] OR ("passive"[All Fields] AND "smoking"[All Fields]) OR "passive smoking"[All Fields]))

**FROM download history:**

| Recent queries in pubmed | |  |  |
| --- | --- | --- | --- |
| Search | Query | Items found | Time |
| **#8** | **Search ((tuberculosis) AND treatment outcome) AND ((((smoking) OR smoking cessation) OR cigarette smoking) OR passive smoking)** | **171** | **6:03:36** |
| #7 | Search (((smoking) OR smoking cessation) OR cigarette smoking) OR passive smoking | 267478 | 6:03:12 |
| #6 | Search passive smoking | 15456 | 6:02:51 |
| #5 | Search cigarette smoking | 46001 | 6:02:25 |
| #4 | Search smoking cessation | 39269 | 6:02:09 |
| #3 | Search smoking | 263940 | 6:02:00 |
| #2 | Search treatment outcome | 1202553 | 6:01:38 |
| #1 | Search tuberculosis | 250243 | 6:01:22 |

**II. Google scholar search**

Keywords used: Tuberculosis Smoking OR smoker OR cigarette OR tobacco "treatment outcome"

**III. Cochrane search**

-Search Name:

Date Run: 26/02/2019 00:23:22

Comment:

ID Search Hits

#1 MeSH descriptor: [Tuberculosis] explode all trees 1981

#2 MeSH descriptor: [Treatment Outcome] explode all trees 125766

#3 ("smoking"):ti,ab,kw (Word variations have been searched) 24727

#4 **#1 and #2 and #3 9**

**One is systematic review and thus omitted**

**IV. Other sources** (snowball searching, conference reviews)
