# Supplementary material for: Effect of smoking on tuberculosis treatment outcomes: A systematic review and meta-analysis
Source: PLoS One. 2020 Sep 17;15(9):e0239333. doi: 10.1371/journal.pone.0239333 (PMC7498109; doi:10.1371/journal.pone.0239333)
Supplement: S1 Table — (DOCX) [file pone.0239333.s003.docx]

| Author | Selection (4) | Comparability (2) | Exposure/outcome (3) | Total score out of 9 |
| --- | --- | --- | --- | --- |
| Leiw,et al | **** | ** | *** | 9 |
| Leung,et al | **** | ** | *** | 9 |
| Salami,et al | **** | ** | *** | 9 |
| Magee,et al | *** | ** | *** | 8 |
| Przybylski,et al | *** | ** | *** | 8 |
| Bonacci,et al | **** | ** | *** | 9 |
| Yamana,et al | **** | ** | *** | 9 |
| Wang,et al | **** | ** | *** | 9 |
| Gegia,et al | **** | ** | *** | 9 |
| Chiang,et al | *** | ** | *** | 8 |
| Maruza,et al | *** | * | *** | 7 |
| Masjedi, et al |  |  |  | Clinical trial |
| Roy,et al | **** | ** | *** | 9 |
| Alo,et al | ** | - | *** | 5 |
| Ma,et al | **** | - | *** | 7 |
| Silva,et al | **** | - | *** | 7 |
| Pazarli,et al | **** | - | *** | 7 |
| Rathee,et al | **** | - | *** | 7 |
| Awaisu, et al |  |  |  | Quasi-experimental |
| Tabaris,et al | ** | - | ** | 4 |
| Reed, et al | **** | ** | *** | 9 |
| Tachfouti, et al | **** | ** | *** | 9 |

**S1 Table.** **Risk of bias assessment of the included studies using the Newcastle-Ottawa quality assessment scale**
