# Supplementary material for: Effect of smoking on tuberculosis treatment outcomes: A systematic review and meta-analysis
Source: PLoS One. 2020 Sep 17;15(9):e0239333. doi: 10.1371/journal.pone.0239333 (PMC7498109; doi:10.1371/journal.pone.0239333)
Supplement: S2 Table — (DOCX) [file pone.0239333.s004.docx]

| **LogOR** | **coefficient** | **Standard error** | **t** | **P > l t l** | **95% Confidence Interval** | **I-squared** |
| --- | --- | --- | --- | --- | --- | --- |
| Year | 0.0024 | 0.0241 | 0.10 | 0.92 | -0.049 - 0.053 | 64.63% |
| High income | -0.2663 | 0.2641 | -1.01 | 0.33 | -0.826 - 0.294 |  |
| Upper-middle income | -0.1700 | 0.2609 | -0.65 | 0.53 | -0.723 – 0.383 |  |
| _constant | -4.1722 | 48.3974 | -0.09 | 0.93 | -106.770 – 98.426 |  |
|  | Joint F test (3, 16) = 0.37; p=0.78  Adjusted R-squared = -37.04% | | | | |  |

**S2 Table**. **Meta-regression of the study timing and income category on the effect of smoking on tuberculosis treatment outcomes**
