# Supplementary material for: Effect of smoking on tuberculosis treatment outcomes: A systematic review and meta-analysis
Source: PLoS One. 2020 Sep 17;15(9):e0239333. doi: 10.1371/journal.pone.0239333 (PMC7498109; doi:10.1371/journal.pone.0239333)
Supplement: S3 Table — (DOCX) [file pone.0239333.s005.docx]

| **Classic fail-safe N** |  |
| --- | --- |
| Z-value for observed studies | 10.33 |
| The P-value for observed studies | 0.00 |
| Alpha | 0.05 |
| Tails | 2.00 |
| Z for alpha | 1.96 |
| Number of observed studies | 20.00 |
| Number of missing studies that would bring p-value to > alpha | 536.00 |
| **Orwin’s fail-safe N** |  |
| The odds ratio in observed studies | 1.396 |
| The criterion for a ‘trivial’ odds ratio | 1.100 |
| Mean odds ratio in missing studies | 1.000 |
| Number missing studies needed to bring odds ratio under 1.1 | 50.000 |

**S3 Table**. **Orwin’s Fail-Safe N analysis for the effect of smoking on poor tuberculosis treatment outcomes**
