# Supplementary material for: Effect of smoking on tuberculosis treatment outcomes: A systematic review and meta-analysis
Source: PLoS One. 2020 Sep 17;15(9):e0239333. doi: 10.1371/journal.pone.0239333 (PMC7498109; doi:10.1371/journal.pone.0239333)
Supplement: S1 Text — (DOC) [file pone.0239333.s006.doc]

| Section/topic | # | Checklist item | Reported on page # |
| --- | --- | --- | --- |
| TITLE | | |  |
| Title | 1 | Effect of smoking on tuberculosis treatment outcomes: A systematic review and meta-analysis | 1 |
| ABSTRACT | | |  |
| Structured summary | 2 | **Introduction**: Numerous studies have explored an effect of cigarette smoking on tuberculosis treatment outcomes but with dissimilar conclusions.  **Objective**: To determine the effect of cigarette smoking on tuberculosis treatment outcomes  **Methods**: PubMed, Cochrane library and Google scholar databases were searched last on February 27, 2019. We applied the random-effects model for the analysis. Publication bias was assessed using funnel plot and Egger’s regression. Furthermore, we performed Orwin’s Fail-Safe N and cumulative meta-analysis to check for small studies’ effect.  **Results**: Out of 22 studies we included in the qualitative synthesis, 12 studies reported p-values less than 0.05 where smoking significantly favored poor treatment outcomes. The remaining 10 studies reported p-values larger than 0.05 implying that smoking does not affect the treatment outcomes. Twenty studies met the criteria for inclusion in a meta-analysis. The meta-analysis found that smoking significantly increased the likelihood of poor tuberculosis treatment outcomes by 51% (OR = 1.51; 95% CI = 1.30 to 1.75 and I-square = 75.1%). In a sub-group analysis, the effect was higher for low- and middle-income countries (OR = 1.74; 95% CI = 1.31 to 2.30) and upper-middle-income economies (OR = 1.52; 95% CI = 1.16 to 1.98) than for high-income ones (OR = 1.34; 95% CI = 1.03 to 1.75) even though the differences in the effects among the strata were not statistically significant as demonstrated by overlapping of confidence intervals of the effects. Meta-regression analysis, adjusted for income economies, found the effect of smoking has not significantly improved over the years (p = 0.92) and thus implying neither of the covariates were source of the heterogeneity.  Egger’s regression test indicated that publication bias is unlikely (p=0.403).  **Conclusion**: Cigarette smoking is significantly linked with poor tuberculosis treatment outcomes | 2&3 |
| INTRODUCTION | | |  |
| Rationale | 3 | One of the reasons that have driven us to conduct this systematic review and meta-analysis was that there were numerous studies done on the effect of cigarette smoking on tuberculosis treatment outcomes but with dissimilar conclusions. Furthermore, previous reviews of poor treatment outcomes of tuberculosis especially in relation to tobacco were limited to mortality, treatment failure or non-conversion of sputum culture. However, this review has additionally considered loss-to follow-up as an outcome to be included in the meta-analysis because tobacco smoking causes a lack of adherence to anti-tuberculosis treatment. | 3 & 4 |
| Objectives | 4 | The objective of this systematic review and meta-analysis was to assess the effect of cigarette smoking on poor tuberculosis treatment outcomes | 4 |
| METHODS | | |  |
| Protocol and registration | 5 | Protocol was developed but not submitted for registration |  |
| Eligibility criteria | 6 | The PICO acronym (Participants; Interventions/exposures; Comparators and Outcomes) was used to serve as a reminder to develop search terms for electronic database search. In this specific review;   - Participants in the studies were tuberculosis patients who were receiving anti-TB treatment - The intervention was smoking cessation or no history of smoking by the participant - The controls are tuberculosis patients who are smokers or have exposure to smoking - Outcomes are tuberculosis treatment outcomes   The eligibility criteria for inclusion of studies into the qualitative synthesis were whether the study assessed TB treatment outcome, and compared TB treatment outcomes between smokers and non-smokers. In addition to the criteria for qualitative synthesis, studies were required to have frequencies for 2x2 table or have sufficient information to extract frequencies for 2x2 table to enable computation of odds ratio in the meta-analysis. There was no restriction by year. However, only studies written in English language were considered because of other language inability by the authors. | 4,5 & 6 |
| Information sources | 7 | PubMed, Cochrane library and google scholar databases were searched using free-text and Medical Subject-Heading (MeSH) terms for tuberculosis, treatment outcome and smoking without the restriction of the study period. Advanced searching was made by combining the search terms using Boolean operators. The last day the electronic database search conducted was on February 27, 2019. Additionally, snowball searching was performed by screening the reference list of studies that are eligible for the review by electronic search. | 5 & 6 |
| Search | 8 | The following search terms were used for the PubMed’s database search:  (("tuberculosis"[MeSH Terms] OR "tuberculosis"[All Fields]) AND ("treatment outcome"[MeSH Terms] OR ("treatment"[All Fields] AND "outcome"[All Fields]) OR "treatment outcome"[All Fields])) AND (((("smoking"[MeSH Terms] OR "smoking"[All Fields]) OR ("smoking cessation"[MeSH Terms] OR ("smoking"[All Fields] AND "cessation"[All Fields]) OR "smoking cessation"[All Fields])) OR ("cigarette smoking"[MeSH Terms] OR ("cigarette"[All Fields] AND "smoking"[All Fields]) OR "cigarette smoking"[All Fields])) OR ("tobacco smoke pollution"[MeSH Terms] OR ("tobacco"[All Fields] AND "smoke"[All Fields] AND "pollution"[All Fields]) OR "tobacco smoke pollution"[All Fields] OR ("passive"[All Fields] AND "smoking"[All Fields]) OR "passive smoking"[All Fields])) | Supporting information (S1 annex) |
| Study selection | 9 | Records that were duplicates were removed using EndNote software. Records that were not relevant to the topic under review, like guidelines, reviews, position papers and those didn’t have abstract (only titles available) were also removed through screening the titles and abstracts.  The eligibility criteria for inclusion of studies into the qualitative synthesis were whether the study assessed TB treatment outcome, and compared TB treatment outcomes between smokers and non-smokers. In addition to the criteria for qualitative synthesis, studies were required to have frequencies for 2x2 table or have sufficient information to extract frequencies for 2x2 table to enable computation of odds ratio in the meta-analysis. | 6 |
| Data collection process | 10 | Data were extracted into an excel sheet by A.B. and B.D, independently. The authors resolved disagreements on the eligibility of a study by discussing and reaching mutual consensus. Studies’ authors were contacted to obtain information on incomplete data but none could provide justifying that the requested data were old and can’t be easily identified from the archive. | 6 & 7 |
| Data items | 11 | The data extraction form consisted study’s author, year, country, design, proportion of HIV positive participants, type of TB assessed (drug-susceptible or drug-resistant or mixed), proportion of participating children aged less than 15 years, the assessed categories poor TB treatment outcomes (cured, treatment completed, treatment failure, loss to follow-up, dead), mean age of smokers, mean age of non-smokers, type of exposure to smoking (active cigarette smoking or passive smoker), frequency of poor outcome among smokers, frequency of treatment success among smokers, frequency of poor outcome among non-smokers, frequency of treatment success among non-smokers, and effect of measure used by the study and its p-value. In this review, while extracting frequencies of outcomes and exposures, ex-smokers or previous-smokers who stopped smoking 6 weeks before initiation of anti-TB treatment were counted as non-smokers | 6 & 7 |
| Risk of bias in individual studies | 12 | The quality of non-randomized studies has been assessed using the Newcastle Ottawa Quality Assessment Scale. This scale assigns a “star” in which a study is judged on three broad perspectives: the selection of the study groups; the comparability of the groups; and the exposure for case-control or cross-sectional studies, or the outcome for cohort studies. The maximum score was 9 points. A score of <5 was considered low quality, 5–7 was considered medium quality, and >7 was considered high quality. Accordingly, studies rated to score of less than 5 stars out of 9 were carefully seen if they have a significant influence on summary effect size. For the randomized clinical trial study, the Cochrane Collaboration’s tool for assessing the risk of bias was used to assess the methodological quality. The Joanna Briggs Institute (JBI) Critical Appraisal Checklist for non-randomized experimental studies was used to assess the risk of bias with the quasi-experimental study. | 7 |
| Summary measures | 13 | Odds ratio was used for the summary measure | 6 |
| Synthesis of results | 14 | The qualitative synthesis was reported by describing the characteristics of the studies. A preponderance of the quantitative statistical analyses was executed using Stata software version 14 by installing meta-package in it. Few of the analyses like Orwin’s Fail-Safe N that Stata doesn’t execute were done employing Comprehensive Meta-Analysis (CMA) version 3 software.  The random-effects model was applied for the quantitative analysis and odds ratios (ORs) of individual studies with their 95% confidence intervals, including the summary odds ratio, were displayed using forest-plot. The studies were assessed for heterogeneity using I-squared and the p-value for Q-statistic. | 8 |

Page 1 of 2

| Section/topic | # | Checklist item | Reported on page # |
| --- | --- | --- | --- |
| Risk of bias across studies | 15 | Publication bias has been assessed by using a funnel plot which is inherently subjective, Egger’s test of significance of bias, and contour-enhanced funnel plot successively. Additionally, trim and fill analysis which iterates computation of estimate effect size until symmetry of funnel plot reaches has been done to check for the effect of publication bias if any on shifting the unadjusted summary effect size. Finally, a small study effect was checked by running cumulative meta-analysis which is also a kind of sensitivity analysis. Orwin’s Fail-Safe N was also done. | 7 |
| Additional analyses | 16 | Sensitivity analysis was done to see whether there is a substantial difference in the reached conclusion on the summary effect size and determine how robust the summary odds ratio was by omitting each study turn by turn. Subgroup analyses were also performed by study countries’ threshold of income economies, the period of the studies and HIV infection status. Reasons for heterogeneity were further explored applying meta-regression analysis on the year of studies adjusting for income level. | 8 |
| RESULTS | | |  |
| Study selection | 17 | A total of 190 records (171 from PubMed/MEDLINE, 9 from Cochrane library and 8 from Google Scholar) were retrieved after the last search on February 27, 2019. Of these, 177 remained for further evaluation after duplicates were removed. By screening the records by their titles and abstracts, 127 records were removed because of the following reasons; their topics are unrelated, found to be guidelines, position paper, review, or there was only a title that was found without abstract. Out of the 50 articles screened, 22 qualified the eligibility criteria and included in the qualitative synthesis. Twenty-eight articles were excluded from the analysis because 18 of them haven’t measured TB treatment outcomes, 6 of them measured the outcome only before follow-up time completed (checking sputum culture conversion at 2 months following initiation of the treatment but comparing the outcomes among smokers and non-smokers), 2 were extended follow-up studies after the initial follow-up completed, 1 article was watermarked as retracted and 1 article’s full text couldn’t be accessed. Of those 22 articles eligible for qualitative synthesis, 20 articles were recruited for meta-analysis while 2 were excluded . The reasons for the ineligibility of the 2 articles for the meta-analysis were; frequencies for cells of 2x2 tables couldn’t be obtained.  Additional records identified through other sources (n =2)  (Records identified through database searching (n = 188)  PubMed (n=171)  Cochrane (n=9)  Google Scholar (n=8)  **Identification**  Records after duplicates removed (n = 177)  Records screened (n = 50)  Records excluded (n =127)  Full-text articles assessed for eligibility (n =22)  Full-text articles excluded, with reasons (n =28)  Outcome of interest not measured (n=24)  After-treatment course completed cohort follow-ups (n=2)  Retracted (n=1)  Full text inaccessible (n=1)  Studies included in qualitative synthesis (n =22)  Studies included in quantitative synthesis (meta-analysis) (n = 20)  **Screening**  **Eligibility**  **Included**  Excluded from meta-analysis with reason (n=2)  Frequencies for 2x2 could not be generated  **Fig 1. Flow chart for selecting studies for the systematic review and meta-analysis for effect of smoking on TB treatment outcomes** | 9 |
| Study characteristics | 18 | Twenty-two studies were found eligible for the qualitative synthesis as shown in the Table below. The study period of the studies ranged from 1999 to 2014 except the study by Reed, et al which didn’t mention its study period but its year of publication was 2013. Nevertheless, the publication year for some studies is as recent as 2019. The designs used to conduct the studies were cohort for 10 studies.  **Table 1. Characteristics of studies included in the review and values of their effect measures with the corresponding p-values**   | **Author** | **Study period** | **Study country** | **Study design** | **Measured Poor outcome** | **Effect**  **measured by** | P-value reported | Sample size | | --- | --- | --- | --- | --- | --- | --- | --- | | Leiw,et al | 2012 | Malaysia | Cross-sectional | LTFU or Failure or Death or TO | aOR | 0.011 | 21426 | | Leung,et al | 2001-2003 | Hong-Kong | Cohort | LTFU or Failure or Death | aOR | 0.001 | 15891 | | Salami,et al | 1991-1999 | Nigeria | Cross-sectional | LTFU or Failure or Death | COR | 0.001 | 1530 | | Magee,et al | 2009-2012 | Georgia | Cohort | LTFU or Failure or Death or TO | aRR | >0.05 | 1321 | | Przybylski,et al | 2001-2010 | Poland | Cross-sectional | LTFU or Failure or Death | COR | 0.930 | 1997 | | Bonacci,et al | 1995-2010 | Mexico | Cohort | LTFU or Failure or Death | aOR | 0.026 or 0.2* | 1022 | | Yamana,et al | 2010-2013 | Japan | Cross-sectional | Death | aOR | 0.028 or 0.123** | 762 | | Wang,et al | 2002-2003 | Taiwan | Cross-sectional | LTFU or Failure or Death | aHR | >0.05 | 523 | | Gegia,et al | 2011-2013 | Georgia | Cohort | LTFU or Failure or Death | aRR | <0.050 | 524 | | Chiang,et al | 2001-2003 | Taiwan | Case-control | LTFU or Failure or Death | aOR | 0.047 or 0.073*** | 302 | | Maruza,et al | 2007-2009 | Brazil | Cohort | LTFU | aOR | 0.007 | 273 | | Masjedi,et al | 2012-2014 | Iran | Clinical trial | LTFU or Failure or Death | COR | 0.001 or 0.07**** | 334 | | Roy,et al | 2011 | India | Case-control | LTFU | aOR | 0.720 | 158 | | Alo,et al | 2010-2012 | Fiji Island | Cross-sectional | LTFU or Failure or Death | COR | 0.500 | 375 | | Ma,et al | 2008-2011 | China | Cross-sectional | Death or Failure | chi2-test | 0.076 | 791 | | Silva,et al | 2005-2007 | Brazil | Cohort | Death | COR | 0.570 | 140 | | Pazarli,et al | 2000-2005 | Turkey | Cohort | LTFU or Failure or Death | Chi2-test | 0.190 | 103 | | Rathee,et al | 2010-2011 | India | Cohort | LTFU or Failure or Death | Proportion | 0.000 | 101 | | Awaisu,et al | 2008-2009 | Malaysia | Quasi-experimental | LTFU or Failure | Chi2-test | 0.043 | 86 | | Tabarsi,et al | 2004-2007 | Iran | Cross-sectional | LTFU or Failure or Death | Proportion | Not reported | 111 | | Reed, et al  ^π^ | Not stated^Ψ^ | Republic of Korea | Cohort | Death | aHR | 0.29 or 0.14***** | 657 | | Tachfouti, et al  ^π^ | 2004-2009 | Morocco | Cohort | Failure | aOR | 0.030 | 727 | | aOR = adjusted odds ratio, COR = Crude odds ratio, aRR = adjusted risk ratio  aHR = adjusted hazard ratio, LTFU = Loss to follow-up, TO = transferred out  ^Ψ^published in 2013  ^π^Not eligible for meta-analysis  *P-value of 0.026 for heavy (≥11cigarette/day) & 0.2 for light smokers (<11 cigarette/day)  **P-value of 0.028 for >50packs/year smokers and 0.123 for ≤ 50packs/year smoker  ***P-value of 0.047 for >20 cigarette/day smokers and 0.073 for 1-20/day  ****P-value of 0.001 for smoker and 0.07 for quitters at treatment initiation  ***** P-value of 0.29 for <1 pack/day smokers and 0.14 for ≥ 1 pack/day smokers | | | | |  | | | | 10 & 11 |
| Risk of bias within studies | 19 | The overall scores for risk of bias assessment of the observational studies according to the Newcastle-Ottawa scale were 7 and greater except for two cross-sectional studies; one conducted in Iran and the other in Fiji. The clinical trial and quasi-experimental studies were assessed based on Cochrane collaboration quality assessment and JBI checklists, respectively as shown in Table 2 below.  Table 2. Risk of bias assessment for the included studies using the Newcastle-Ottawa quality assessment scale   | Study’s first author | Selection (4) | Comparability (2) | Exposure/outcome (3) | Total score out of 9 | | --- | --- | --- | --- | --- | | Leiw,et al | **** | ** | *** | 9 | | Leung,et al | **** | ** | *** | 9 | | Salami,et al | **** | ** | *** | 9 | | Magee,et al | *** | ** | *** | 8 | | Przybylski,et al | *** | ** | *** | 8 | | Bonacci,et al | **** | ** | *** | 9 | | Yamana,et al | **** | ** | *** | 9 | | Wang,et al | **** | ** | *** | 9 | | Gegia,et al | **** | ** | *** | 9 | | Chiang,et al | *** | ** | *** | 8 | | Maruza,et al | *** | * | *** | 7 | | Masjedi, et al |  |  |  | Clinical trial | | Roy,et al | **** | ** | *** | 9 | | Alo,et al | ** | - | *** | 5 | | Ma,et al | **** | - | *** | 7 | | Silva,et al | **** | - | *** | 7 | | Pazarli,et al | **** | - | *** | 7 | | Rathee,et al | **** | - | *** | 7 | | Awaisu, et al |  |  |  | Quasi-experimental | | Tabarsi,et al | ** | - | ** | 4 | | Reed, et al | **** | ** | *** | 9 | | Tachfouti, et al | **** | ** | *** | 9 | | 10 |
| Results of individual studies | 20 | Twenty studies were included for the meta-analysis. The smallest sample size was 86 and the study with a sample size of 21,426 was the largest study included in the analysis . The number of participants of the studies added up to be 47,770. Participants who were smokers accounted for 33% of the total participants. TB treatment outcomes were poor for 21% of the total study participants. Fig 2 demonstrates the odds ratios of individual studies with their respective confidence intervals and weights    **Fig 2. Forest plot of odds ratios (ORs) displaying the effect of smoking on TB treatment outcomes and heterogeneity of studies** | 17 |
| Synthesis of results | 21 | The combined (summary) odds ratio showed that patients with poor TB treatment outcomes were significantly exposed to smoking by more than 50% than patients with treatment success (OR 1.51, 95% CI 1.30-1.75). Heterogeneity was measured using I-squared and it indicates that about 75% of the observed variability among the odds ratios of the studies was attributed to true heterogeneity between studies (I-squared = 75.1%, P = 0.000). | 17 |
| Risk of bias across studies | 22 | To minimize the subjectivity of interpreting the funnel plot as symmetric, a statistical test of symmetry called Egger’s test was performed and found that there was no statistically significant association between studies’ effect sizes and their standard error as the p-value is insignificant ( Egger’s bias coefficient = 0.52, P = 0.403).  In addition to the funnel plot and Egger’s test, the contour enhanced funnel plot was used to assess the area where the missing studies are perceived. Small studies and large studies that reported smoking significantly decrease the likelihood of poor tuberculosis treatment outcomes are missing on the contours of the statistical significant area on the left side of the plot. Nevertheless, small studies that were reporting there is no statistically significant association are not missing.  Furthermore, the robustness of the combined odds ratio was evaluated using the trim and fill analysis. Accordingly, the unbiased/adjusted odds ratio from the random-effects model was found to be 1.47 (95% CI = 1.26 to 1.71) and the unadjusted/observed one was 1.51 (95%CI = 1.30 to 1.75).  Orwin’s Fail-Safe N method was also used to determine how many missing studies would bring the overall effect of 1.51 to a value that would represent the smallest effect of 1.10 (selected by the researchers). In the running analysis below, Orwin’s Fail-Safe N is 50, suggesting that there would need to be 50 studies with a mean odds ratio of 1.0 added to the analysis before the cumulative effect would become trivial (defined as the odds ratio of 1.10). The classic (Rosenthal’s) fail-safe N, which computes the number of hidden studies required to make the effect not statistically significant, is 536.  To investigate if publication bias operates mainly on smaller studies, an analysis that is restricted to larger studies was done. The 20 studies were sorted from most precise to least precise which roughly corresponds to largest to smallest sample size. With the 11 largest studies in the analysis, the cumulative odds ratio is 1.51. With the addition of another 9 smaller studies, the point estimated doesn’t consistently shift to any side, and the odds ratio remains 1.51.  It is also noticeable that the analysis that incorporates all 20 studies assigns 80.7% of its weight to the first 11 larger studies implying that smaller studies are given less weight which prevents their ability to introduce bias (see figures 7-9). | 20-22 |
| Additional analysis | 23 | **Sensitivity analysis**  Sensitivity analysis was done to investigate the influence of each individual study on the overall summary odds ratio by omitting each study turn by turn and re-estimating the summary odds ratio. As shown in Fig 3 below, there is no single study for which point estimate of its omitted analysis (small circle) that lies outside of the confidence interval of the combined meta-analysis represented by the solid vertical lines. Rather the point estimates of omitted analysis for the entire included studies cluster around the point estimate of the combined meta-analysis (middle solid vertical line).    **Subgroup analysis**  Sub-group analysis was performed to investigate the possibility that the effect of smoking on poor tuberculosis treatment outcomes may vary by income economies of countries in which the studies were undertaken. The analysis found out that smoking was significantly associated with poor tuberculosis outcomes irrespective of the threshold of income economies of the study countries. The odds ratios were 1.74 (95% CI = 1.31-2.30), 1.52 (95% CI = 1.16-1.96) and 1.34 (95% CI = 1.03-1.74) for lower-middle, upper-middle and high income economies, respectively. In the sub-group analysis, heterogeneity of between studies’ odds ratios for lower-middle, upper-middle and high income countries were found decreased from the crude I-squared of 75.1% (P = 0.00) to I-squared values equal to 64.4% (P = 0.024), 52.2% (P = 0.027) and 73.6% (P = 0.004), respectively. There was no study retrieved from lower-income countries to be included in this sub-group analysis.  Additionally, subgroup analysis was done based on the period the studies were conducted. The period of study was classified as after 2010 and before or during 2010. Accordingly, even though the effect of smoking on TB treatment outcomes favored poor outcomes in both periods, studies conducted during 2010 and before demonstrated high between-study heterogeneity (I-squared = 75.9%, P = 0.000). But the variability of odds ratio between studies conducted after 2010 was smaller (I-square = 53.1%, P =0.029).  To further investigate the source of heterogeneity, subgroup analysis was done by the HIV infection status of the study participants of individual studies. In doing so, those 5 studies that didn’t report the HIV status of their participants were excluded and only studies that reported the proportion of HIV infection among their participants were analyzed. From the subgroup analysis, it was found that there was insignificant heterogeneity between studies conducted on HIV uninfected population (I-squared = 13.8%, P = 0.324). However, heterogeneity remained significantly high between studies conducted on mixed study participants of HIV infected and uninfected (I-squared = 83.0%, P = 0.000), and between those studies conducted on HIV infected participants (I-squared = 70.1%, P = 0.000). Meta-regression The year that the study was undertaken and the income economy of the country of the study were fit to meta-regression model to investigate whether these study-level covariates have driven the heterogeneity. Lower-middle income was used as a reference category. The joint test for both covariates gave a p-value of 0.78, indicating evidence for no association of at least one of the covariates. More than 64% (I-squared residual = 64.63%) of the observed variation of odds ratios of the effect of smoking among the studies was attributed to between-study variations. The negative adjusted R-squared (-37.04%) implies the covariates explain less of the heterogeneity that would be expected even by chance alone (S2 Table). | 16-18 |
| DISCUSSION | | |  |
| Summary of evidence | 24 | This systematic review and meta-analysis aimed to evaluate the association between smoking and TB treatment outcomes. We identified no study that reported smoking significantly favors successful TB treatment outcomes. Instead our pooled estimate of odds ratios found that active cigarette smoking is significantly associated with poor TB treatment outcomes. High heterogeneity suggests we should be cautious in generalizing the pooled mean odds ratio estimate to different populations . This considerable statistical heterogeneity in odds ratios between studies could arise from methodological or clinical diversity or, in all likelihood, a combination of both. The review included studies with diverse designs including experimental, cohort, case-control and cross-sectional studies . Clinical diversity is also likely because studies included were diverse in their participants’ characteristics (HIV status, TB drug susceptibility), the TB treatment outcomes measured, study context (country income category) and study periods .  Contrarily, a meta-analysis by Samuels JP et al showed that there is no difference between smokers and non-smokers. However, the fact that their analysis was based on studies involving only MDR/XDRTB patients may be the reason for our differences .  In this meta-analysis, pooling changed the significance of effects originally found in individual studies from significant to not significant and vice versa. The studies by Bonnaci et al and Yamana et al were reporting significant p-values but their effects were no longer significant in the meta-analysis. The loss of significance resulted from aggregating light smokers and heavy smokers together as smokers instead of comparing them separately with non-smokers in the original studies. Similarly, ex-smokers and current smokers were independently compared with never-smokers in Leung et al but ex-smokers (“an ever smoker who had stopped smoking for at least for 1 year before the current TB episode”) were merged with non-smokers in this meta-analysis under the assumption that 1 year offers sufficient recovery time from the effect of smoking . On the other hand, Magee et al’s originally non-significant p-value for adjusted effect size shifted to significance in the meta-analysis (as defined by confidence intervals that do not contain the null value OR of 1). This could be explained by a larger sample size (i.e. crude frequency that is not unadjusted for different factors) and smaller p-value (tendency to be significant) effect .  We ran a sensitivity analysis to investigate the influence of each individual study on the overall summary estimate that found that omitting any of the studies had no excessive influence on the summary odds ratio .  We investigated sources of heterogeneity based first on country income category. Doing so reduced the level of heterogeneity to moderate level implying that some variability between studies was attributable to context even if not statistically significant. Second, we explored whether timing of study implementation before or during 2010 or after 2010 explained any variability between studies and found less variability in later than earlier studies. We also concluded that diversity in HIV status among study participants was responsible for heterogeneity in the effect of smoking where studies conducted on participants not living with HIV had insignificant heterogeneity. We explain the significant heterogeneity in studies involving people living with HIV on the variability in immunity levels in these populations depending on clinical stages of their disease .  Finally, by adjusting for country income category and timing of the studies, our meta-regression analysis sought to assess whether the effect of smoking decreased over time in conjunction with progressive reductions in nicotine content of cigarettes . However, we learned that the effect of smoking on TB treatment outcomes neither significantly changed overtime nor showed difference among study countries’ income category. Therefore, it was unlikely that the year of study or the income category of studies’ countries caused the heterogeneity. Our analyses also indicate that publication bias was unlikely. | 21-23 |
| Limitations | 25 | This review has relied entirely on searching free electronic study databases. Thus it is likely we missed studies indexed in health sciences databases like Embase. Using odds ratios as the measure for pooling of effect sizes where the magnitude of poor TB treatment outcome was not a rare event (21%) and approximating a risk ratio is another limitation of this review. The search language restricted to English may have led to missing additional work published in other languages. | 23 |
| Conclusions | 26 | Smoking is significantly linked with poor tuberculosis treatment outcomes, particularly in lower-middle-income and upper-middle-income countries as compared to high-income countries though the difference was not statistically significant. | 23 |
| FUNDING | | |  |
| Funding | 27 | There was no source of funding for this systematic review and meta-analysis. |  |

**REFERENCES**

1. Reed GW, Choi H, Lee SY, Lee M, Kim Y, Park H, Lee J, Zhan X, Kang H, Hwang S *et al*. Impact of diabetes and smoking on mortality in tuberculosis. *PloS one* 2013, 8(2):e58044.

2. Tachfouti N, Nejjari C, Benjelloun MC, Berraho M, Elfakir S, El Rhazi K, Slama K. Association between smoking status, other factors and tuberculosis treatment failure in Morocco. *The international journal of tuberculosis and lung disease : the official journal of the International Union against Tuberculosis and Lung Disease* 2011, 15(6):838-843.

3. Liew SM, Khoo EM, Ho BK, Lee YK, Mimi O, Fazlina MY, Asmah R, Lee WK, Harmy MY, Chinna K *et al*. Tuberculosis in Malaysia: predictors of treatment outcomes in a national registry. *The international journal of tuberculosis and lung disease : the official journal of the International Union against Tuberculosis and Lung Disease* 2015, 19(7):764-771.

4. Leung CC, Yew WW, Chan CK, Chang KC, Law WS, Lee SN, Tai LB, Leung EC, Au RK, Huang SS *et al*. Smoking adversely affects treatment response, outcome and relapse in tuberculosis. *The European respiratory journal* 2015, 45(3):738-745.

5. Salami AK, Oluboyo PO. Management outcome of pulmonary tuberculosis: a nine year review in Ilorin. *West African journal of medicine* 2003, 22(2):114-119.

6. Magee MJ, Kempker RR, Kipiani M, Tukvadze N, Howards PP, Narayan KM, Blumberg HM. Diabetes mellitus, smoking status, and rate of sputum culture conversion in patients with multidrug-resistant tuberculosis: a cohort study from the country of Georgia. *PLoS One* 2014, 9(4):e94890.

7. Przybylski G, Dabrowska A, Trzcinska H. Alcoholism and other socio-demographic risk factors for adverse TB-drug reactions and unsuccessful tuberculosis treatment - data from ten years' observation at the Regional Centre of Pulmonology, Bydgoszcz, Poland. *Medical science monitor : international medical journal of experimental and clinical research* 2014, 20:444-453.

8. Bonacci RA, Cruz-Hervert LP, Garcia-Garcia L, Reynales-Shigematsu LM, Ferreyra-Reyes L, Bobadilla-del-Valle M, Canizales-Quintero S, Ferreira-Guerrero E, Baez-Saldana R, Tellez-Vazquez N *et al*. Impact of cigarette smoking on rates and clinical prognosis of pulmonary tuberculosis in Southern Mexico. *The Journal of infection* 2013, 66(4):303-312.

9. Yamana H, Matsui H, Fushimi K, Yasunaga H. Treatment options and outcomes of hospitalised tuberculosis patients: a nationwide study. *The international journal of tuberculosis and lung disease : the official journal of the International Union against Tuberculosis and Lung Disease* 2015, 19(1):120-126.

10. Wang JY, Hsueh PR, Jan IS, Lee LN, Liaw YS, Yang PC, Luh KT. The effect of smoking on tuberculosis: different patterns and poorer outcomes. *The international journal of tuberculosis and lung disease : the official journal of the International Union against Tuberculosis and Lung Disease* 2007, 11(2):143-149.

11. Gegia M, Magee MJ, Kempker RR, Kalandadze I, Chakhaia T, Golub JE, Blumberg HM. Tobacco smoking and tuberculosis treatment outcomes: a prospective cohort study in Georgia. *Bull World Health Organ* 2015, 93(6):390-399.

12. Chiang YC, Lin YM, Lee JA, Lee CN, Chen HY. Tobacco consumption is a reversible risk factor associated with reduced successful treatment outcomes of anti-tuberculosis therapy. *International journal of infectious diseases : IJID : official publication of the International Society for Infectious Diseases* 2012, 16(2):e130-135.

13. Maruza M, Albuquerque MF, Coimbra I, Moura LV, Montarroyos UR, Miranda Filho DB, Lacerda HR, Rodrigues LC, Ximenes RA. Risk factors for default from tuberculosis treatment in HIV-infected individuals in the state of Pernambuco, Brazil: a prospective cohort study. *BMC Infect Dis* 2011, 11:351.

14. Masjedi MR, Hosseini M, Aryanpur M, Mortaz E, Tabarsi P, Soori H, Emami H, Heidari G, Dizagie MK, Baikpour M. The effects of smoking on treatment outcome in patients newly diagnosed with pulmonary tuberculosis. *The international journal of tuberculosis and lung disease : the official journal of the International Union against Tuberculosis and Lung Disease* 2017, 21(3):351-356.

15. Roy N, Basu M, Das S, Mandal A, Dutt D, Dasgupta S. Risk factors associated with default among tuberculosis patients in Darjeeling district of West Bengal, India. *Journal of family medicine and primary care* 2015, 4(3):388-394.

16. Alo A, Gounder S, Graham SM. Clinical characteristics and treatment outcomes of tuberculosis cases hospitalised in the intensive phase in Fiji. *Public Health Action* 2014, 4(3):164-168.

17. Ma Y, Che NY, Liu YH, Shu W, Du J, Xie SH, Li L. The joint impact of smoking plus alcohol drinking on treatment of pulmonary tuberculosis. *European journal of clinical microbiology & infectious diseases : official publication of the European Society of Clinical Microbiology* 2019.

18. Silva DR, Menegotto DM, Schulz LF, Gazzana MB, Dalcin Pde T. Factors associated with mortality in hospitalized patients with newly diagnosed tuberculosis. *Lung* 2010, 188(1):33-41.

19. Pazarlı P, Duman DY, Moçin ÖY, Karagöz T. The effect of smoking on treatment outcome of multidrug-resistant tuberculosis. *Turkish Thoracic Journal* 2013, 14:93-97.

20. Rathee D, Arora P, Meena M, Sarin R, Chakraborty P, Jaiswal A, Goyal M. Comparative study of clinico-bacterio-radiological profile and treatment outcome of smokers and nonsmokers suffering from pulmonary tuberculosis. *Lung India : official organ of Indian Chest Society* 2016, 33(5):507-511.

21. Awaisu A, Nik Mohamed MH, Mohamad Noordin N, Abd Aziz N, Syed Sulaiman SA, Muttalif AR, Ahmad Mahayiddin A. The SCIDOTS Project: evidence of benefits of an integrated tobacco cessation intervention in tuberculosis care on treatment outcomes. *Subst Abuse Treat Prev Policy* 2011, 6:26.

22. Tabarsi P, Chitsaz E, Moradi A, Baghaei P, Farnia P, Marjani M, Shamai M, Amiri M, Nikaein S, Mansouri D *et al*. Treatment outcome, mortality and their predictors among HIV-associated tuberculosis patients. *International journal of STD & AIDS* 2012, 23(9):e1-4.

23. Higgins JP, Thompson SG, Deeks JJ, Altman DG. Measuring inconsistency in meta-analyses. *BMJ (Clinical research ed)* 2003, 327(7414):557-560.

24. Pildal J, Hrobjartsson A, Jorgensen KJ, Hilden J, Altman DG, Gotzsche PC. Impact of allocation concealment on conclusions drawn from meta-analyses of randomized trials. *International journal of epidemiology* 2007, 36(4):847-857.

25. Gagnier JJ, Morgenstern H, Altman DG, Berlin J, Chang S, McCulloch P, Sun X, Moher D. Consensus-based recommendations for investigating clinical heterogeneity in systematic reviews. *BMC medical research methodology* 2013, 13:106.

26. Samuels JP, Sood A, Campbell JR, Khan FA, Johnston JC. Comorbidities and treatment outcomes in multidrug resistant tuberculosis: a systematic review and meta-analysis. *Scientific reports* 2018, 8(1):1-13.

27. Arcavi L, Benowitz NL. Cigarette smoking and infection. *Arch Intern Med* 2004, 164(20):2206-2216.

28. Hersey P, Prendergast D, Edwards A. Effects of cigarette smoking on the immune system. Follow-up studies in normal subjects after cessation of smoking. *The Medical journal of Australia* 1983, 2(9):425-429.

29. Kim J, Bang H. Three common misuses of P values. *Dental hypotheses* 2016, 7(3):73-80.

30. Tobias A. Assessing the influence of a single study in the meta-analysis estimate. *Stata Tech Bull* 1999, 47:15-17.

31. World Bank Country and Lending Groups. [https://datahelpdesk.worldbank.org/knowledgebase/articles/906519-world-bank-country-and-lending-groups]

32. Marshall MM, McCormack MC, Kirk GD. Effect of cigarette smoking on HIV acquisition, progression, and mortality. *AIDS education and prevention : official publication of the International Society for AIDS Education* 2009, 21(3 Suppl):28-39.

33. World Health Organization. Global nicotine reduction strategy. *Geneva, Switzerland: WHO* 2015.
